# Ohno-miRNAs: miRNA pairs derived from whole-genome duplication

**DOI:** 10.1371/journal.pcbi.1013766

**Published:** 2025-12-03

**Authors:** Leonardo Agasso, Ivan Molineris, Michele Caselle

**Affiliations:** 1 Department of Physics, University of Turin and INFN, Turin, Italy; 2 Department of Life Sciences and Systems Biology, University of Turin, Turin, Italy; Johns Hopkins University School of Medicine, UNITED STATES OF AMERICA

## Abstract

Two rounds of whole-genome duplication (WGD) occurred about 500 million years ago and played a major role in the evolution of the vertebrate genomes. Human genes derived from WGD are called “ohnologs”. Ohnologs are involved in fundamental biological processes and significantly contributed to the complexity of the human gene regulatory network. Given the central role of miRNAs in gene regulation, we investigated the contribution of ohnolog miRNAs (ohno-miRNAs) to the human gene regulatory network. We identified intragenic ohno-miRNAs as having higher retention rates compared to intragenic Small Scale Duplicated (SSD) miRNAs. Ohno-miRNAs also show high sequence similarity, a stronger tendency to regulate common target genes and are typically more expressed compared to miRNAs unrelated to WGD events. Analyzing the role of ohno-miRNAs in the human gene regulatory network, we showed that ohno-miRNAs are statistically overrepresented in specific network motifs commonly associated with redundancy and complexity, highlighting their central role in gene regulation.

## Author summary

MicroRNAs (miRNAs) are short molecules that regulate gene expression at the post-transcriptional levels, inhibiting mRNA transcription. As protein-coding genes, miRNAs can evolve through duplication of small segments of the genome (Small Scale Duplication, SSD) or Whole Genome Duplication (WGD). Two WGD events occurred at the beginning of the vertebrate lineage, approximately 500 million years ago, increasing the number of miRNAs as well as that of protein-coding genes. In this work, we analyzed the effects the two WGD events on the human miRNA repertoire, with a particular focus on how they reshaped the regulatory interactions between miRNAs and mRNAs. We observed how miRNAs originated from WGD (ohnolog miRNAs) are more conserved and tend to be involved in different regulatory circuits known to have a role in shaping the complexity of regulatory networks. In line with previous results obtained on protein-coding genes, our results suggest a central role of ohnolog miRNAs in the emergence of regulatory complexity in vertebrate genomes.

## Introduction

Gene duplication is the evolutionary process in which a region of DNA hosting a gene produces one or more copies that are temporarily relieved of selective pressure and may potentially develop adaptations for new functions over time. Small-scale duplication (SSD) events include all processes that duplicate small portions of a genome, usually a single gene or a small set of genes. Since SSDs induce small changes in the genotype, the results on the phenotype are usually of limited magnitude. Whole-genome duplication (WGD), often referred to as “polyploidization”, is a process of genome duplication that generates additional copies of the entire genome. WGD events provide raw genetic material to facilitate phenotypic evolution and drastically increase genome complexity. Because of their extreme nature, whole genome duplication (WGD) events are almost exclusively evolutionary dead ends, as they typically involve sudden and dramatic phenotypic changes and are likely to have an immediate impact on the fitness of the organism, compromising both its fertility and short-term survival. Notwithstanding this, WGD, although rare, played major roles in evolution. In particular, the occurrence of two rounds of WGD at the beginning of the vertebrate lineage is now widely accepted, as first proposed by Susumu Ohno in his book “Evolution by Gene Duplication” [1]. In fact, WGD events can provide immediate evolutionary benefits to the affected lineage by inducing successful responses to abrupt changes in the environment and can significantly boost the size and complexity of the impacted genome, causing beneficial effects in the long term [2,3]. In the human genome, approximately 20-30% of the protein-coding genes can be traced back to WGD events [4]. Thanks to a detailed reconstruction of the evolutionary tree of the vertebrate lineage, a reliable list of putative pairs of ohnolog genes in vertebrates was recently proposed in [5] and [6]. Ohnolog genes show distinctive features that help shed light on the mechanisms behind ohnolog gene retention. In mammals, they undergo fewer small-scale duplications and are refractory to copy number variation compared to SSD-derived genes, suggesting stronger dosage balance constraints [5]. Ohnologs are often linked to disease susceptibility. Singh et al. [7] showed that genes prone to autosomal-dominant or gain-of-function mutations, such as oncogenes and dominant disease genes, were preferentially retained after the two vertebrate WGDs. Their relevance in different cancer types is supported by the observation that ohnolog genes are enriched in components relevant to various types of cancer, such as melanoma, B-cell lymphoma, lung adenocarcinoma, and others [8]. In addition, Makino and McLysaght showed that ohnologs are more likely to be essential (i.e., their removal results in a lethal or sterile phenotype) than SSD-derived paralogues. They are strongly associated with monogenic disorders and diseases linked to dosage sensitivity, as evidenced by their overrepresentation among OMIM disease genes. Notably, 75% of candidate genes implicated in Down Syndrome are ohnologs [5]. All these results appear to be at odds with the expected back-up role of duplicate genes, which should provide a buffer against such effects, and are regarded as evidence that ohnologs predominantly consist of dosage-balanced genes [5]. From a functional, non-pathological perspective, ohnologs are found to be more frequently involved in signaling, development, and transcriptional regulation [9] and are enriched in Gene Ontology categories associated with the general level of complexity of the organism [10]. From a gene expression point of view, the gene expression profile and subcellular localization display more divergence between the two members of a WGD pair than between the members of an SSD one [9]. Whole genome duplication has also been shown to affect the structure of human gene regulatory networks: motif analysis of such networks indicates that the two rounds of WGD have largely contributed to regulatory redundancy, promoted synergy between different regulatory layers, and generated motifs that are usually associated with complex functions [11]. One of the goals of the present paper is to improve our understanding of the role played by WGD in shaping the vertebrate regulatory network by including the miRNA layer in the analysis.

MicroRNAs (miRNAs) are a class of small, endogenous, single-stranded, non-coding RNAs (ncRNAs) that play important roles in regulating eukaryotic gene expression, mainly at the post-transcriptional level [12]. In their mature form, miRNAs are 19-25 nucleotides in length and regulate gene expression by binding to the 3’ untranslated regions (UTRs) of target messenger RNAs (mRNAs), leading to translational repression or mRNA degradation. The miRNA-mRNA interaction is combinatorial: a single miRNA can directly target hundreds of mRNAs, whereas a single mRNA can be targeted by multiple miRNAs. The binding is strongly dominated by a short subsequence of the mature miRNA, the so-called “seed region” [13], usually consisting of 6–8 nucleotides, mostly situated at positions 2-8, from the 5’ end. More than 60% of human protein-coding genes harbor predicted miRNA target sites [14], and more than half of the human protein-coding genome is estimated to be regulated by miRNAs [15]. Aberrant miRNA expression is known to play an important role in pathological processes, including allergic and autoimmune diseases [16–18]. The lists of WGD-derived genes reported in [5,6] include only protein-coding genes. Recently, however, a list of inferred WGD-derived pairs of miRNAs (hereafter referred to as ohno-miRNAs) has become available [19,20]. This enables a more comprehensive study of the regulatory interactions that arise from WGD events. In particular, we focus on the role of WGD in shaping post-transcriptional regulation. We show that several of the features already observed in the gene regulatory network at the transcriptional level [11] are also present at the post-transcriptional level, and that ohno-miRNAs are involved in a set of complex network motifs that appear to be specifically associated with their WGD origin.

## Materials and methods

### SSD-derived and WGD-derived gene pairs

Following Mottes et al. [11] we obtained the WGD-derived pairs of ohnolog genes by merging the results of [5] with the list of human ohnologs available from the OHNOLOGS v2 database [6]. From the OHNOLOGS v2 database, we retained only the pairs meeting the *strict* criteria for high-confidence ohnologs. Only protein-coding genes were considered. The list of human protein-coding paralogue gene pairs was obtained from the Ensembl database [21]. We removed from this list all the pairs that were identified as WGD-derived. In order to ensure compatibility among different datasets, all the genes were traced back to their corresponding occurrences in the comprehensive gene annotation of the GENCODE database [22]. Following these criteria, we identified 9,348 WGD-derived pairs of protein-coding genes, involving 7,775 single genes, and 122,863 SSD-derived pairs of protein-coding genes, involving 13,784 single genes. Considering single genes, 6,047 genes are involved both in WGD-derived pairs and in SSD-derived pairs.

### Ohno-miRNAs and SSD-derived miRNA pairs

To retrieve a reliable list of WGD-derived (ohno-miRNAs) and SSD-derived miRNA pairs for our analyses, we developed a custom public pipeline leveraging annotations from [19] and the standardized miRNA nomenclature of MirGeneDB [20]. ohno-miRNA pairs were obtained from the data in [19], which identifies miRNA pairs and families originating from WGD events in the vertebrate lineage, also providing information on their ancestral subgenomes. Although the data does not explicitly list ohno-miRNA pairs in a usable format, we manually parsed the original data to allow our pipeline to project these ancestral WGD families onto individual genomes (e.g., human). This allowed us to reconstruct the set of ohno-miRNAs in multiple vertebrate genomes of interest, provided that they are annotated in MirGeneDB. To ensure interoperability with downstream analysis, our pipeline links each MirGeneDB miRNA entry to its corresponding Ensembl identifier, as well as the miRBase name and accession number where these are available. This mapping is essential to proceed with the analysis using external resources such as TarBase and MirDIP, which have not yet adopted the MirGeneDB nomenclature to identify miRNAs. Similarly, SSD-derived pairs were obtained from MirGeneDB, leveraging their nomenclature and excluding pairs already identified as WGD-derived. This procedure led to 114 ohno-miRNA pairs and 2,466 SSD-derived miRNA pairs. As an alternative, Ensembl is a valid resource for SSD-derived miRNA pairs. We report results based on MirGeneDB-derived SSD pairs in the main text. A full replication of the analyses using Ensembl SSD pairs (1,131 miRNA pairs) is reported in S1 Text. In order to perform the stratification of duplicate pairs between pre-2R and post-2R, we used MirGeneDB to obtain the Last Common Ancestor (LCA) of the two miRNAs. We considered pre-2R all those LCAs older than Vertebrata.

### Independent detection of intragenic duplicate miRNA pairs

To validate and possibly expand the established lists of ohno-miRNAs, we adopted an independent gene-based procedure to identify intragenic ohno-miRNAs. Leveraging annotated WGD-derived and SSD-derived protein-coding gene pairs, we identified those where both genes host a miRNA. using BEDTools [23] and filtering for miRNAs recognized as *bona fide* by MirGeneDB [20]. Only pairs in which both miRNAs conserve the strand orientation relative to their host genes were retained. To avoid artificially-inflated pair counts, in cases where one or both host genes hosted multiple miRNAs, we applied a reciprocal best alignment hit (RBH) procedure, retaining only pairs that were mutual best hits after computing sequence similarity for each possible miRNA pair across a duplicate gene pair. For example, in the ohnolog pair DNM2 and DNM3, DNM3 hosts both MIR199A2 and MIR214, but only MIR199A1–MIR199A2 met the RBH criterion and was retained. Additionally, we manually classified MIR196A1-MIR196A2 as a ohnolog pair. Although GENCODE annotations initially placed these miRNAs on SSD host genes (i.e., HOXB7-HOXC6), further inspection revealed that this classification relied on non-basic transcripts. Given their conserved position within HOX clusters, we treated them as intragenic ohno-miRNAs for downstream analysis.

**Fig 1:**
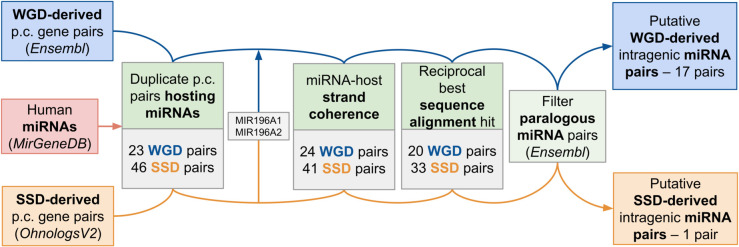
Intragenic duplicate pairs retrival. Schematic overview of the pipeline used to identify putative intragenic ohno-miRNA and SSD-derived miRNA pairs. The number of putative pairs retained at each step is reported.

This procedure is intended primarily as a check of existing datasets (e.g., [19]), but it revealed two additional high-confidence ohno-miRNA pairs that are currently not annotated (i.e., MIR499A-MIR208A and MIR499A-MIR208B). This procedure is detailed in the graphical scheme in Fig 1.

### Aligning miRNA sequences

The list of human miRNAs is retrieved from the GENCODE database. In order to obtain every mature miRNA originating from each miRNA gene, we leveraged data from miRBase v22 [24] and Ensembl. Through miRBase, it was also possible to obtain the sequences for each mature miRNA and each pri-miRNA originating from a miRNA gene of interest. Given a putative ohno-miRNA pair, we want to assign a similarity score based on the sequences. To do so, we retrieved every mature miRNA originating from a miRNA gene and aligned it with every other mature miRNA originating from the duplicate miRNA. The sequence similarity assigned to the ohno-miRNA pair is the highest score obtained using this procedure.

**Fig 2:**
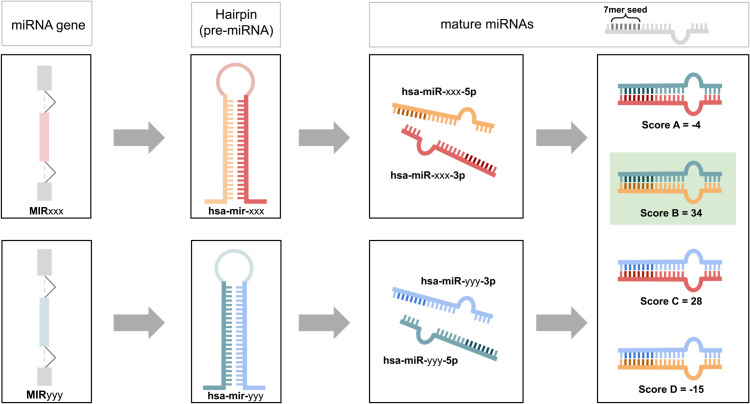
MiRNA alignment procedure. Schematic representation of how the alignment score is assigned to miRNA gene pairs, leveraging mature miRNA alignments. Mature miRNAs are aligned using the modified version of the Needleman-Wunsch algorithm. The represented case would see the pair MIRxxx and MIRyyy assigned an alignment score of 34.

The alignment score was established using a modified version of the Needleman-Wunsch algorithm, where the match and mismatch were assigned greater (or lower, in the case of mismatches) weight in the substitution matrix, outlined in Fig 2. The seed was considered to be formed by nucleotides from 2 to 8 in a 7mer seed, starting numbering at the 5’ end of the mature miRNA. Matches and mismatches not involving nucleotides in the seed are given a weight of ±1, while for in-seed matches and mismatches the weight is assigned to be ± 5. The results are consistent considering different lengths of the seed (*6mer*, *8mer*) and different weights assigned to in-seed matches and mismatches (±3, ±4, ±6).

### miRNA-target interaction networks

The miRNA-target networks used to assess motif enrichment and target similarity come from the TarBase [25] and the miRDIP [26] databases. TarBase is limited to experimentally validated interactions, while miRDIP integrates miRNA-target interactions from different databases and prediction methods. Both networks are biased, but in opposite directions. Literature-based collections like TarBase are characterized by numerous missing interactions (false negatives); they are biased towards genes that have received more attention from the scientific community. As pointed out in the Introduction, WGD-derived genes are often associated with diseases and organism complexity, which are preferential subjects of published papers. On the other hand, networks based on *in silico* predictions of the interactions, such as MirDIP, are typically characterized by a large number of false positives. Notwithstanding these differences, we shall see in the following that the two networks lead essentially to the same enrichment patterns (see Fig C, D and E S1 Text). We consider this observation to be strong evidence of the overall robustness of our results.

The procedures used to obtain suitable networks from these databases are partially borrowed from [11]. The TarBase network was constructed by selecting all the interactions from Normal/Primary cell lines or tissues (excluding “cancer” and “other” categories), with positive evidence for a direct interaction between the miRNA and the target gene. Moreover, we only kept those interactions that were reported to have been obtained from a high-throughput approach. The resulting network comprises 713 miRNAs and 10,458 target genes, combined into 102,774 interactions. The miRDIP network was processed by leveraging the presence of an integrative score assigned by the authors to each miRNA-target pair. It is well established that the interactions provided by a miRNA-target prediction program are often noisy (false positives or biologically irrelevant) [27]. To overcome this problem, we decided to keep only the interactions belonging to the “Very high” score class (top 1% of interactions in the database). In this case, the resulting network is larger and is composed of 1,847 miRNAs and 15,738 targets combined in 465,874 interactions. Since TarBase and miRDIP report data related to mature miRNAs, each mature miRNA was traced back to its miRNA gene of origin in order to build a direct network involving miRNA genes instead of mature miRNAs (see Methods). The resulting networks are very similar in terms of nodes, with 713 miRNAs and 9,161 genes common between TarBase and MirDIP; however, they differ in terms of the interactions, as only 9,809 edges are common between the two networks. We provide a summary of the presence of ohnologs and paralogues in the two networks in Tables G-J in S1 Text.

### Protein-protein interaction networks

Consistent with [11], protein-protein interactions were retrieved from two distinct databases: PrePPI [28] and STRING [29]. From PrePPI, we retained only the high-confidence interactions with experimental validation from the HINT database or the APID database. The STRING database was processed by selecting a high confidence score (>960) to keep the sizes of the two PPI-networks comparable. The protein IDs were mapped to the corresponding gene names using the UniProtKB Database [30].

We ended up with 45,386 PPIs and 8,944 genes in the PrePPI network, while the resulting STRING network is composed of 51,268 PPIs and 9,758 genes. The two networks largely overlap in terms of nodes (6,730 genes are present in both networks) but differ in terms of interactions, with only 16,044 interactions in common. As for the miRNA-target interaction network, we provide a summary of the presence of ohnologs and paralogues in the two employed networks in Tables K and L in S1 Text.

### Sequence identity between duplicate miRNAs requires manual curation of the miRNA-target networks

When looking at enrichment scores, a potential problem with the TarBase and MirDIP databases is that, in some cases, duplicate miRNA genes can result in identical mature miRNAs after the processes of transcription and cleavage and are treated by the databases as a single miRNA. Some examples of this redundancy can be found in the following miRNA pairs: the MIR196A1 and MIR196A2 genes are both transcribed and cleaved into the same 5’ mature miRNA named hsa-miR-196a-5p [31]. A similar case is the pair MIR218-1 and MIR218-2; they have different 3’ mature miRNAs (hsa-miR-218-1-3p and hsa-miR-218-2-3p) but an identical 5’ mature miRNA (hsa-miR-218-5p). To address this issue, we manually curated the regulatory networks constructed from the two databases to maintain the distinction between the miRNAs that were lost in the databases. In particular, we mapped each mature miRNA back to its miRNA gene, thus mapping the original “mature miRNA-mRNA” network into a “miRNA gene-mRNA” network. Whenever a mature miRNA originates from more than one miRNA gene, the new network has two different corresponding nodes. Whenever this procedure produces two duplicate miRNAs that are indistinguishable (i.e., they have the exact same connections), the pair is excluded from the subsequent analyses.

### Sørensen-Dice similarity coefficient

As a metric for the interaction similarity of two duplicate miRNAs, we used the Sørensen-Dice similarity coefficient, defined in the following way:


$$S(A,B)=\frac{2|A\cap B|}{\left|A|+|B\right|}$$

Where *A* and *B* are the sets of target genes of the two miRNAs in the pair. The Sørensen-Dice similarity coefficient is equal to 0 when there are no target genes in common between the two ohno-miRNAs and is equal to 1 when all interactions are in common.

### MiRNA expression datasets

Tissue-specific miRNA expression data were obtained from two up-to-date, high-confidence resources: MirGeneDB 3.0 [20] and MiRNATissueAtlas v3 [32]. These two datasets provide comprehensive insights into the expression of miRNAs across tissues in *Homo sapiens*. MiRNATissueAtlas is a large-scale dataset that collects expression data for nine classes of different non-coding RNAs across a wide range of tissues. We downloaded the expression matrices for mature miRNA expression in Homo sapiens. In cases where multiple mature isoforms were derived from the same precursor, their expressions were summed. When computing cosine similarity between pairs, count values were first normalized within each sample by dividing each entry by the total number of transcripts observed in that sample. An identical procedure was used to parse the MirGeneDB expression matrix [20]. Each duplicate miRNA is labeled as “WGD” (ohno-miRNAs) or “SSD” according to its duplication origin. In cases where a miRNA underwent both WGD and SSD events in its evolutionary history, we labeled it as WGD to keep the two sets disjoint. This resulted in the analysis of 112 ohno-miRNAs and 172 SSD-derived miRNAs from MiRNATissueAtlas, as well as 112 ohno-associated miRNAs and 177 SSD-derived miRNAs from MirGeneDB. To quantify differences in normalized expression levels, we computed pairwise distances as 1−Cosinesimilarity between the expression vectors of each miRNA across all samples in the MiRNATissueAtlas. We used this dataset due to its comprehensive coverage and the large number of available tissue samples.

### Null models

To assess motif enrichment, we introduced an ensemble of 1,000 null models by applying random rewiring to our networks. We employed a degree-preserving procedure for randomization (as in [11]), preserving the degree for each node while randomly rewiring interactions between miRNAs and target genes. This ensured that every gene and miRNA maintained the same number of interactions as in the original network. By doing so, we aimed to investigate whether the observed enrichment patterns were influenced by degree-degree correlations within the duplicate pairs, as these correlations remained consistent in the randomized network set.

### Quantifying ohno-miRNA enrichment in different network motifs

Network motifs refer to specific patterns of nodes and edges that exhibit a notable overrepresentation in the regulatory network when compared to randomized networks [33]. It is widely accepted that these motifs have undergone positive evolutionary selection due to their functional efficacy. To evaluate the enrichment of network motifs, we present the Z-score associated with the motif count within the ensemble of randomized networks. The Z-score calculation is generally given by:


$$Z=\frac{n-{\overset{―}{n}}_{null}}{\sigma_{null}}$$

where *n* is the motif count on the real network, ${\overset{―}{n}}_{null}$ is the average motif count computed across 1,000 randomized networks, and $\sigma_{null}$ is the standard deviation derived from the same distribution. We present the Z-scores computed for the single pair (or single miRNA in the case of PPI-delta). Differences from the standard way of evaluating the Z-scores are explained in Fig M, in S1 Text. We plotted only the Z-score of intragenic ohno-miRNA pairs recognized as actual duplicate pairs by Ensembl, since the set of SSD duplicate pairs was downloaded from Ensembl. If ${\overset{―}{n}}_{null}\neq 0$ and $\sigma_{null}=0$ (i.e., at least one motif is present in the real network, but we cannot find a motif involving the pair in any of the 1,000 randomized networks), the pair is discarded because it would be impossible to evaluate the Z-score. On the other hand, if ${\overset{―}{n}}_{null}=0$ and $\sigma_{null}=0$, we set *Z* = 0. Discarded pairs never exceed 1% of the total pairs in a given set. However, since these cases involve pairs relevant to the analysis, we report the discarded pairs in Tables E and F in S1 Text. Pairs in which one or both miRNAs were not present in the interaction network were discarded. When analyzing delta motifs, individual miRNAs belong to both WGD and SSD pairs: for example, MIR10A is in a WGD pair with MIR10B according to our analysis and is in an SSD pair with MIR125A according to Ensembl. These miRNAs, which are present in both the WGD and SSD sets, are labeled as WGD.

## Results

**Fig 3:**
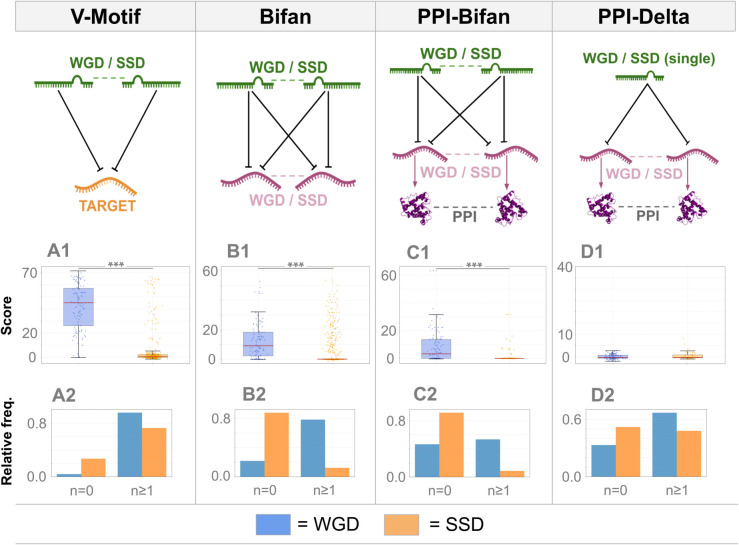
Comparative analysis of regulatory motifs among WGD and SSD miRNA pairs. V-Motif (A1-A2), Bifan (B1-B2), PPI-Bifan (C1-C2), and PPI-Delta (D1-D2). Panels A1-D1 show the distribution of enrichment scores computed for each miRNA-target pair (WGD pairs on the left, SSD pairs on the right). Panels A2-D2 display the number of motif instances collected in classes: For V-motifs (A2), pairs are binned by the number of motifs (0, 1–50, >50); for all other motifs (B2, C2, D2), pairs (or single miRNAs in D2) are separated in two classes, corresponding to being involved in 0 or ≥1 motif. An enrichment signal relative to WGD pairs is observed for V-Motifs, Bifan, and PPI-Bifan. In contrast, PPI-Delta motifs show minimal differences, with nearly all miRNAs showing no enrichment.

As mentioned above, the main goal of this paper is to study the combined post-transcriptional and transcriptional regulatory motifs involving WGD-derived genes and miRNAs, with the aim of better understanding the role of WGD in shaping the vertebrate regulatory network. The first section of the results is devoted to this task, focusing in particular on the so-called V, Delta and Bifan motifs (see Fig 3 for a pictorial description of these motifs). We then turn to a set of properties that distinguish ohno-miRNAs from other miRNAs, which may shed light on their specific roles and significance. In particular, we examined the enrichment of ohno-miRNAs within WGD-derived host genes relative to SSD hosts. We showed that ohno-miRNAs are more conserved than SSD pairs and are typically more highly expressed. We also discussed some overrepresented Gene Ontology terms specifically associated with ohno-miRNAs. Together, these results point to a specific role and importance of ohno-miRNAs, which we further discuss in the Discussion.

### Ohno-miRNAs are enriched in specific network motifs within the regulatory network

Leveraging data from [19], it is possible to obtain a reliable list of ohno-miRNA pairs in several vertebrate genomes. Equipped with the full list of ohno-miRNAs in the human genome, we can now study the role they play in the human regulatory network. This study is the natural continuation of the analysis in ref. [11], wherein the role of WGD transcription factors in shaping the human regulatory network was highlighted. In [11] it was shown that the ohnolog pairs of transcription factors were involved in a few specific regulatory motifs that were detected by studying their enrichment in the regulatory network with respect to suitable null models; the aim of this section is to extend this analysis to miRNAs. To address this challenge, we used two different miRNA-target networks, TarBase [25] and MirDIP [26], and two different protein-protein interaction networks, PrePPI [28] and STRING [29]. The results obtained with the TarBase and PrePPI databases are reported in the main text, while in Fig E in S1 Text, we present those obtained with MirDIP and STRING. Comparable results are obtained even when using distinct reference datasets that share only limited overlap (see Discussion).

#### The outdegree distribution of ohno-miRNAs slightly differs from that of ordinary miRNAs.

**Fig 4:**
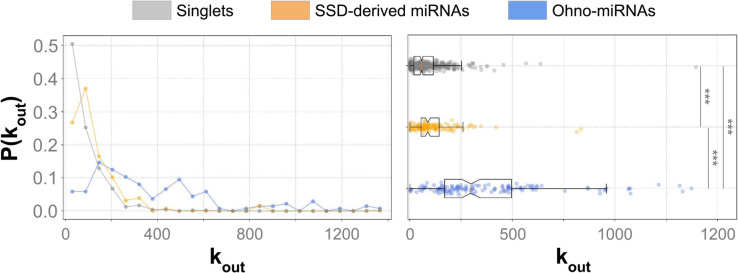
Outdegree distributions. Outdegree distributions of ohno-miRNAs compared with SSD-derived miRNAs, and miRNAs that didn’t undergo any duplication (*singlets*), in the TarBase network. (***: *p* < 0.001, Kolmogorov-Smirnov test).

We analyzed the outdegree distribution of the ohno-miRNAs present in the TarBase network, comparing it with that of SSD-duplicate miRNAs. In this context, the outdegree corresponds to the number of target genes for each miRNA. In general, duplicate miRNAs tend to have a slightly higher outdegree than non-duplicate ones. This tendency is stronger when considering ohno-miRNAs compared to SSD-derived miRNAs (see Fig 4). This difference is taken into account in subsequent analyses, ensuring that our results are not attributable to the difference in outdegree distribution. Indeed, all the results reported below were obtained by comparing real networks with a null model generated by randomly reshuffling the links while keeping the degrees of the nodes constant (see Methods). Moreover, this approach accounts for biases such as the TarBase tendency to report more targets for genes that have received greater attention from the scientific community. This bias is also controlled by changing the miRNA-mRNA network to MirDIP (see S1 Text).

#### V-motif enrichment in the miRNA-target networks.

The first non-trivial motif we analyzed is the so-called “V-motif” consisting of a duplicate pair of miRNAs interacting with a common target gene. A motif enrichment analysis using a “pairwise” Z-score shows that both SSD and WGD pairs tend to retain a certain number of common targets, highlighting a clear enrichment of V-motifs in the ohno-miRNAs with respect to SSD-derived miRNAs (Fig 3A1 and 3A2). This tendency is similar to what was observed for transcription factors [11]; thus, This signal of “redundancy” in gene regulation appears to be a hallmark of whole-genome duplication (WGD) and is likely linked to the increased complexity of vertebrate regulatory networks compared to those of other organisms [34].

#### Bifan enrichment.

The second regulatory motif we analyzed is the bifan, a structure in which a pair of duplicate miRNAs simultaneously regulate a pair of duplicate target genes (see, for instance, [35] for a discussion of the role and functions of this motif at the transcriptional level). The transcriptional version of this motif, in which a pair of duplicate transcription factors regulates a pair of duplicate targets, was studied in detail in [11]. We evaluated the enrichment of bifans with respect to null models. As for the V-motifs, both the WGD pairs and the SSD pairs are strongly enriched in bifans (Fig 3B1); however, many SSD-derived miRNA pairs are involved in exactly zero bifans (Fig 3B2). This is the main difference between WGD and SSD pairs and explains the increase in the score distribution of WGD bifans with respect to the SSD ones. When considering bifans, the creation of such motifs by SSD requires long evolutionary times and is most likely a two-step process. Initially, a miRNA is duplicated, and the two resulting miRNAs share their targets. If the temporal stoichiometric disequilibrium is overcome, one of these common targets is duplicated, thus generating a bifan that is then conserved. There is a deep difference between WGD and SSD in this perspective: during the WGD event *both* the miRNA and the target are simultaneously duplicated.

A particular instance of a bifan is the case where two duplicate miRNAs target two duplicate transcription factors (TFs). First, we decided to list this subset of bifans as a relevant outcome of our analysis. Among these bifans, many motifs of clear biological relevance may be observed:

- A dense overlapping regulon (i.e., the overlap of two or more bifan structures) involves the three ohno-miRNAs MIR196A1, MIR196A2 and MIR196B, each targeting both HOXB8 and HOXC8. The three miRNAs are hosted on three different HOX loci (MIR196A1 within the HOXB cluster, MIR196A2 within the HOXC cluster, MIR196B within the HOXA cluster). HOX genes are well known for their crucial role in vertebrate development, providing information on the positions of tissues and directing morphogenesis through finely regulated, time-controlled transcriptional activation [36–38]. The post-transcriptional regulatory roles of miRNAs in these processes remain less well understood [31]. The presence of WGD-derived bifans centered on HOX genes and regulated by ohno-miRNAs suggests a potential mechanism for fine-tuning gene expression within critical developmental pathways, contributing to the precision and robustness of HOX gene regulation via miRNA-mediated inhibition.
- Another biologically relevant motif involves the ohno-miRNA pair MIR24-1 and MIR24-2, both targeting the transcription factors SOX7 and SOX18. These two SOX-F genes belong to a family known to regulate vascular and cardiac development and have been shown to act redundantly in arteriovenous identity and cardiovascular morphogenesis, as demonstrated in zebrafish and mouse models [39]. The presence of a WGD-derived bifan regulating SOX7 and SOX18 suggests a possible conserved post-transcriptional mechanism contributing to the robustness of vascular gene regulation during development.

These two examples not only involve a bifan of ohno-miRNAs targeting ohnolog TFs; remarkably, we observed that, in both cases, the two TFs have conserved transcription factor binding sites in Homo sapiens, according to the CIS-BP database (version number 3.0) [40], suggesting potential synergy between the transcriptional and post-transcriptional levels.

#### WGD bifans show a strong enrichment with respect to SSD bifans when the target genes interact at the protein level.

A major finding of our analysis is the strong enrichment of WGD-derived bifans compared to SSD-derived ones when the duplicate target genes are not only duplicates of the same type as the miRNAs (SSD or WGD) but are also involved in protein-protein interactions; we call such bifan a PPI-bifan. Interestingly, more than half of the ohno-miRNA pairs are involved in at least 1 PPI-bifan, compared to only about 5% of the SSD pairs (Fig 3C2). In addition, distributions of enrichment scores (Fig 3C1) with the SSD-derived pairs show a significant difference. These results align with the hypothesis that WGD genes are preferentially retained when stoichiometric constraints are critical for the proteins they encode [41,42], as for proteins interacting within complexes. In contrast, the formation of SSD bifans appears to follow a two-step process that does not inherently account for stoichiometric balance, increasing the likelihood of imbalance when such structures arise.

#### PPI-Delta motifs show no enrichment.

We analyzed the so-called delta motifs, where two duplicate targets of the same miRNA also interact at the protein level. This analysis was performed separately for miRNAs derived from WGD and SSD events, where miRNAs that underwent both WGD and SSD events are considered WGD-derived. No significant enrichment is detected, as depicted in Fig 3D1 and 3D2.

These results suggest that the enrichment we observed relative to PPI-Bifan motifs is linked to the particular topology of the PPI-bifan and not to a generic preference of ohno-miRNAs for duplicate pairs involved in protein-protein interactions. These signals hold across multiple datasets as shown in S1 Text.

#### Subgenome imbalance does not influence network topology.

**Fig 5:**
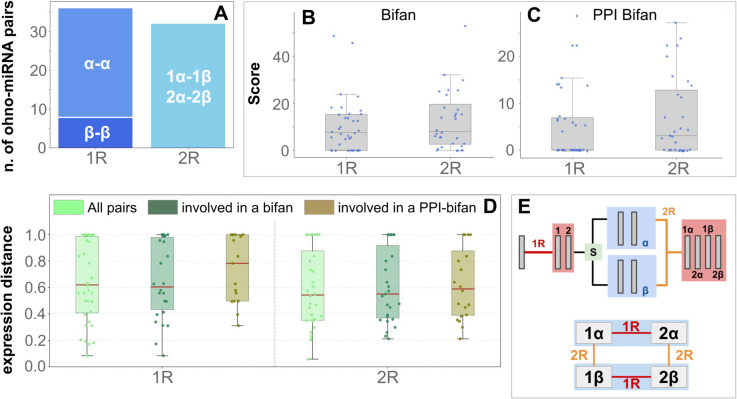
Subgenome identity affects miRNA retention but not motif enrichment or expression correlation. (A) Distribution of WGD-derived miRNA pairs across subgenomes. Pairs derived from the first (1R) and second (2R) rounds of whole-genome duplication are grouped by subgenome identity. As expected from Peterson et al. (2022), *β*–*β* pairs from the 1R are underrepresented due to reduced conservation on the *β* subgenome. (B-C) Enrichment score for simple bifans (B) and PPI-bifans (C) in 1R- and 2R-derived pairs. No significant difference is observed, suggesting that motif enrichment is a general feature of WGD-derived miRNA pairs. (D) Distribution of expression correlations between 1R- and 2R- derived pairs. Expression correlation is measured as *1-Cosine similarity* (see Methods), filtering pairs involved in different motifs. No significant differences in correlations are observed; a slight increase in expression correlation is observed when considering only pairs involved in PPI-bifans. (E) Schematic representation of the 2 WGD events. The scheme is based on [19] and [43], used to classify 1R- and 2R-derived WGD pairs into *α*–*α*, *β*–*β*, *α*–*β* classes.

Peterson et al. (2022) [19] showed that among WGD-derived miRNAs, a strong subgenome imbalance is present, favoring miRNAs originating from subgenome *α* over those from subgenome *β* (*α* and *β* are the two subgenomes that underwent the second round of WGD, an allotetraploidy event, described in [19,43] and depicted in Fig 5E). As shown in Fig 5A, *β*–*β* pairs are underrepresented, consistent with reduced conservation of *β*-subgenome miRNAs. Labeling the subgenome enables the analysis of pairs duplicated in 1R and 2R events. Notably, we did not find a significant difference in the involvement of 1R- vs. 2R-derived pairs in simple bifans or PPI-bifans (Fig 5B and 5C), nor in the distribution of the distances of expression in a pair (Fig 5D). This suggests that, while subgenome identity is extremely informative for understanding single-miRNA evolution, the enrichment of regulatory motifs appears to be a general feature of WGD-derived miRNA pairs.

### Intragenic ohno-miRNAs are a specific outcome of Whole-Genome Duplication

A peculiar feature of WGD-derived miRNA pairs is their enrichment in the introns of WGD-derived host genes with respect to SSD-derived pairs. This observation shows that the evolution of ohno-miRNAs in the human genome was shaped by specific constraints that are different from those of miRNAs originating from small-scale duplications. Our goal in this section is to define and extract intragenic ohno-miRNAs (pairs of putative ohno-miRNAs hosted on ohnolog protein-coding genes) using recent resources on WGD-derived miRNAs in the vertebrate genome [19,20] together with ohnolog gene annotation from the literature [5,6], and to compare them with similar SSD-derived miRNA pairs hosted on SSD-derived protein-coding genes obtained from the Ensembl database (details on the pipeline in Methods). We present here the results for miRNAs recognized as *bona fide* by MirGeneDB 3.0 [20]. This comparison revealed a striking asymmetry in the human genome:

- 17 ohno-miRNA pairs are hosted within ohnolog gene pairs and are recognized as paralogues in both MirGeneDB and Ensembl (plus one additional pair, MIR196A1–MIR196A2, validated manually;
- Only one miRNA pair is hosted within SSD-derived gene pairs and is also recognized as a paralogue pair by at least one of the sources.

We initially identified 20 putative intragenic ohno-miRNA pairs and 33 putative intragenic SSD-derived miRNA pairs. Excluding those pairs that are not recognized as duplicates by MirGeneDB, 17 intragenic ohno-miRNAs and only one SSD-derived pair passed the filter. Two of the three “rejected” putative ohnolog pairs (MIR208A–MIR499B and MIR208B–MIR499B) are plausible ohno-miRNAs that are currently not recognized as duplicates by Peterson et al. (2022) [19] or Ensembl. Their position within the ohnolog MYH genes, together with their high sequence similarity, suggests that they may be a case of misclassification and has prompted us to discuss them in detail (see Discussion).

In summary, our analysis revealed a peculiar signature of WGD duplication events: intragenic duplicate miRNAs hosted on duplicate protein-coding genes are almost exclusively ohnologs. SSDs rarely recreate this arrangement, indicating that this co-retention of host and miRNA could be a hallmark of WGD and may result from precise selective pressure. We list our results in Tables 1 and 2. The miRNA pairs listed in Table 1 are all intragenic *bona fide* ohno-miRNA pairs (i.e., single miRNAs are recognized as such by MirGeneDB and the two miRNAs are known paralogues according to either Ensembl or MirGeneDB); we report a list of unfiltered intragenic ohno-miRNAs in Tables A and B in S1 Text.

**Table 1:** Intragenic ohno-miRNA pairs recognized as duplicates by Ensembl.

Intragenic ohno-miRNA 1	Host gene 1	Intragenic ohno-miRNA 2	Host gene 2
MIR199A1	DNM2	MIR199B	DNM1
MIR199A1	DNM2	MIR199A2	DNM3
MIR199B	DNM1	MIR199A2	DNM3
MIR103A1	PANK3	MIR103A2	PANK2
MIR103A1	PANK3	MIR107	PANK1
MIR103A2	PANK2	MIR107	PANK1
MIR26B	CTDSP1	MIR26A1	CTDSPL
MIR26B	CTDSP1	MIR26A2	CTDSP2
MIR26A1	CTDSPL	MIR26A2	CTDSP2
MIR196A2	HOXC6	MIR196A1	HOXB7
MIR10A	HOXB3	MIR10B	HOXD3
MIR218-1	SLIT2	MIR218-2	SLIT3
MIR204	TRPM3	MIR211	TRPM1
MIR152	COPZ2	MIR148B	COPZ1
MIR128-1	R3HDM1	MIR128-2	ARPP21
MIR153-1	PTPRN	MIR153-2	PTPRN2
MIR33B	SREBF1	MIR33A	SREBF2

**Table 2:** The only pair of intragenic SSD-derived paralogue miRNAs recognized as duplicates by Ensembl.

Intragenic SSD-derived miRNA 1	Host gene 1	Intragenic SSD-derived miRNA 2	Host gene 2
MIR208B	MYH7	MIR208A	MYH6

Interestingly, we observed that intragenic ohno-miRNA are mainly hosted on gene families, including:

- pantothenate kinase (PANK) genes: MIR103A1 (PANK3), MIR103A2 (PANK2), MIR107 (PANK1);
- C-terminal domain small phosphatase (CTDSP) genes: MIR26A1 (CTDSPL), MIR26A2 (CTDSP2), MIR26B (CTDSP1);
- dynamin (DNM) genes: MIR199A1 (DNM2), MIR199A2 (DNM3), MIR199B (DNM1);
- myosin heavy chain (MYH) genes: MIR208A (MYH6), MIR208B (MYH7), MIR499A (MYH7B);
- homeobox (HOX) genes: MIR196A2 (HOXC6), MIR196A1 (HOXB7), MIR10A (HOXB3), MIR10B (HOXD3).

### Ohno-miRNAs are more conserved than SSD-derived pairs

**Fig 6:**
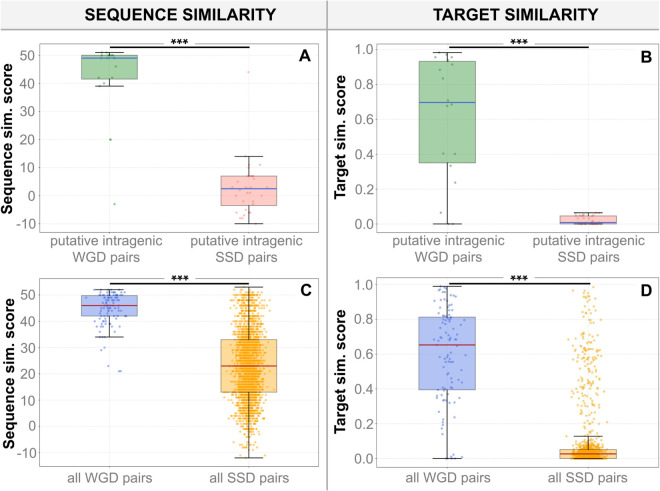
Sequence similarity and target similarity. (A, B) Sequence similarity (A) and target similarity (B); Sørensen–Dice coefficient on TarBase targets for putative intragenic miRNA pairs. (C, D) Sequence similarity (C) and target similarity (D) for the full set of ohno-miRNA pairs and SSD-derived miRNA pairs, as annotated in [19,20]. WGD-associated miRNA pairs consistently show higher scores than SSD-derived pairs across both metrics. (***: *p* < 0.001, Kolmogorov-Smirnov test).

In this section, we asked whether ohno-miRNAs are more conserved than SSD-derived pairs at the molecular level, considering both sequence similarity and target overlap. We selected ohno-miRNA pairs and SSD-derived miRNA pairs in the human genome from the recent literature [19,20], using alignment scores to assess sequence similarity and Sørensen-Dice coefficients over TarBase target sets to assess target similarity (see Methods). Additional comparisons using the MirDIP network are provided in S1 Text. In general, ohno-miRNAs are significantly more similar than SSD-derived pairs in terms of sequence and target mRNAs (Fig 6C and 6D). Interestingly, SSD-duplicate miRNA pairs originating after the two rounds of whole-genome duplication are slightly less similar when compared with all the SSD-duplicate miRNA pairs (see Fig G in S1 Text).

These results also shed light on putative intragenic pairs as well. Almost all the putative intragenic ohno-miRNA pairs display high similarities, whereas SSD-derived intragenic miRNAs are almost exclusively concentrated at very low sequence similarity scores (Fig 6A and 6B). This observation suggests two possible scenarios: (1) all excluded miRNA pairs are not paralogues; thus, they were not duplicated along with their host genes but emerged independently. This scenario is supported by a background expectation: given the number of SSD-derived protein-coding gene pairs and assuming that miRNA presence in host genes is approximately random, we would expect some number of false-positive pairings simply by chance (see S1 Text). (2) These miRNA pairs were duplicated during SSD events but subsequently underwent rapid evolutionary divergence (neofunctionalization), leading to poor sequence similarity and making them difficult to recognize as paralogues.

### Ohno-miRNAs exhibit higher expression than SSD-derived miRNAs

**Fig 7:**
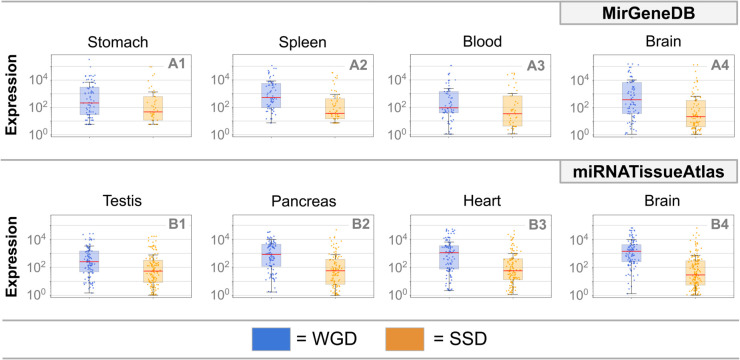
Duplicate miRNA expression. Expression of ohno-miRNAs (blue) and SSD-derived miRNAs (orange) across multiple human tissues, based on two independent datasets: MirGeneDB (A1–A4) and MiRNATissueAtlas (B1–B4). Each panel shows the distribution of miRNA expression within a tissue. Across all tissues, WGD-derived miRNAs consistently exhibit higher median expression levels than SSD-derived miRNAs. MiRNAs that both underwent WGD and SSD are included exclusively in the WGD set.

To understand the links between duplication events and miRNA expression, we compared the expression of ohno-miRNAs with that of pairs derived from SSD events. This analysis is performed on two independent sources: MirGeneDB [20] and MiRNATissueAtlas [32]. The average expressions of miRNAs belonging to the two groups show a marked difference, consistent across tissues and robust to the choice of database (Fig 7: median expression is higher for ohno-miRNAs than for SSD-derived miRNAs; results are consistent across all tissues in both datasets. Further examples are reported in Figs I and J in S1 Text). The fact that the greater structural and functional conservation highlighted in the previous sections is accompanied by higher expression is a hallmark of ohno-miRNAs’ stronger functional constraints and their integration into conserved regulatory programs.

### Gene ontology enrichment of ohno-miRNAs

We performed a gene ontology (GO) enrichment analysis using DAVID [44] to functionally characterize ohno-miRNAs. Importantly, we performed the analysis using all the miRNAs as the background set to avoid biased results that reflect general miRNA functions. This analysis revealed that in the Cellular Component ontology, the most relevant term is “*extracellular vesicle*” (Benjamini-Hochberg corrected $p<6\;\times \;10^{-7}$). In the Biological Process ontology, we found enrichment for a group of terms related to signaling regulation (e.g., “*negative regulation of signaling*”, “*negative regulation of cell communications*”, corrected $p<6\;\times \;10^{-6}$). These results align with the known roles of WGD-derived genes in vertebrate development, suggesting an implication of ohno-miRNAs in tissue organization, intercellular communication, and morphogenesis. As a test of consistency, we tested different combinations of randomly-chosen miRNAs without finding any particular enrichment.

## Discussion

### A simple argument for the ohno-miRNA retention

As reported in the Introduction, ohnolog genes are known to have many peculiar features and are involved in many crucial functions. These results are at odds with the expected backup role of duplicate genes (widely observed in less complex eukaryotes) which should provide a buffer against such effects. Within this context, the hypothesis of a prevalence of dosage-balanced genes among ohnologs has been proposed [5]. Changing the stoichiometry of members of a set of interacting genes (e.g., members of the same protein complex or the same pathway) may affect the function of the whole, resulting in detrimental effects on fitness. This implies a selective pressure that maintains the balance between the dosage of genes in these sets [45,46]. WGD facilitates the maintenance of the stoichiometric balance of all components of a dosage-balanced gene set compared to SSD. Moreover, if any gene is lost after a WGD it is likely to produce a dosage imbalance of the corresponding gene products; this may lead to the preferential retention of dosage-balanced ohnologs [46]. Consistent with this assumption, retained ohnologs have been shown to be enriched for dosage-balanced genes that are resistant to subsequent SSD and copy number variation in human populations [5].

A similar mechanism may explain the predisposition towards ohno-miRNA retention within the human genome. It has been extensively established that a single miRNA typically targets the transcripts of hundreds of genes [47], effectively suppressing their translation [48]. Considering that these target genes are usually dispersed throughout the genome, WGD maintains the appropriate stoichiometric equilibrium between a miRNA and its entire target set, while SSD events may alter these balances. This argument is even stronger when considering intragenic miRNAs, the transcription of which depends on the host gene. It seems that essentially no miRNA-host gene structure has been retained after a small-scale duplication in the vertebrate lineage for more than 500 million years. A consequence of this reasoning is that the presence of a miRNA within a gene, even if it is not in dosage balance, reduces the probability that the gene can undergo an SSD fixed in the population, given that the SSD of the locus would also imply the duplication of the miRNA that is in dosage balance. Our results support this hypothesis, highlighting how SSD-derived gene pairs do not harbor pairs of intragenic SSD-derived miRNAs, with the exception of the peculiar case of MIR208A-MIR208B (see Discussion).

### V-motif enrichment and regulatory redundancy

V-motifs are created straightforwardly through a duplication event; immediately after the duplication, the two miRNAs target the same set of genes. However, this redundancy is only temporary, and over rather short evolutionary timescales, the two miRNAs start to differentiate, following the standard processes of neofunctionalization or subfunctionalization [49,50]). This is clearly visible in Fig 3A2, where most of the SSD pairs have exactly zero V-motifs. On the contrary, the enrichment that we observe in those V-motifs involving ohno-miRNAs (in Fig 3A1) suggests that, for this class of duplicate miRNAs, there is a strong evolutionary pressure to maintain this regulatory redundancy. This is most likely a consequence of the dosage-balance constraint discussed above. This regulatory redundancy, previously observed at the transcriptional level in WGD-derived pairs of Transcription Factors [11], is confirmed at the post-transcriptional level. Regulatory redundancy is known to play an important role in shaping the complexity of the organism [34,51,52]. It increases the robustness of the network against mutations [53] and allows for the implementation of complex regulatory mechanisms, such as the bifans that we shall discuss below. Moreover, due to the different promoters regulating the two miRNAs, redundancy allows their expression to be differentiated temporally or spatially [51]. This redundancy also enables independent tuning of their response to external stimuli [52], while maintaining the regulation of the same target genes.

### Significance and functional roles of bifans in miRNA-target regulatory networks

The bifan is one of the most overrepresented network motifs in gene regulatory networks at the transcriptional level [54,55]. Previous research on the importance of WGD duplications in the topology of regulatory networks highlighted that many motifs are particularly overrepresented when both the regulators and the targets are WGD or SSD genes [11]. As shown above, the same is true at the post-transcriptional level. There are several reasons that may explain the specific importance of miRNA mediated bifans. They allow for finer modulation of the expression of the targets, differentiating (thanks to the different promoters in front of the two miRNAs) the response to external stimuli. More generally, they can be considered as “decision-making” motifs [35]. These same arguments also hold for the transcriptional version of these motifs. What makes the miRNA-mediated version special is that miRNAs are known to act as fine-tuners of gene expression and are particularly effective, thus strongly preserved during evolution, in performing the complex and delicate functions discussed above.

### Synergy between protein-protein interaction and miRNA regulation

One of the most interesting outcomes of our analysis is that genes interacting at the protein level have a higher probability of being co-targeted by a pair of miRNAs in a bifan, and this probability is even higher if both the miRNAs and the targets are ohnolog genes. As we mentioned above, this could be explained by a dosage-balance mechanism driving WGD pair retention, but it is also an indication of the strong synergy between the protein-protein interaction layer and the post-transcriptional layer of regulation. This same synergistic behavior was observed in [11] at the transcriptional level. This is in line with the idea that miRNA regulation acts as a fine-tuning layer of regulation and is thus more immediately related to the stoichiometric constraints imposed by the presence of a protein-protein interaction between the two targets.

### The case of MIR208A-MIR208B

**Fig 8:**
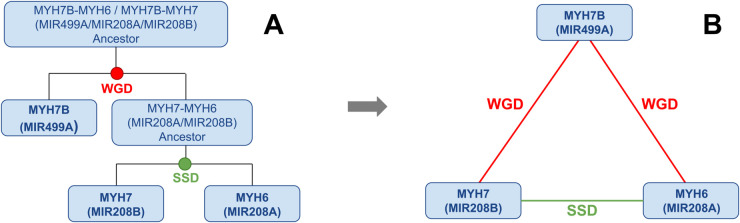
Evolution of MIR499A, MIR208A and MIR208B. Suggested evolutionary history linking MYH7B, MYH7 and MYH6 and their intronic miRNAs (MIR499A, MIR208A, MIR208B). As explained in the Discussion, the duplication times suggested by Ensembl are considered to be errors in the annotations.

The only pair of duplicate miRNAs hosted on a pair of SSD-derived genes is MIR208A (on MYH6), MIR208B (on MYH7); it is interesting to address this case in more detail. The two host genes are long-known paralogue genes [56,57]. According to Ensembl, their last common ancestor was in the clade of *Opisthokonta*, while the last common ancestor of MIR208A and MIR208B is reported to be in the clade of *Euteleostomi*. Thus, according to Ensembl, the duplication of the two miRNAs would have occurred after the duplication of their host genes and after the two rounds of WGDs. However, this scenario is likely due to an annotation inconsistency. Our hypothesis is that after the WGD event that generated the pair composed of MIR499A and the MIR208A-MIR208B ancestor with the respective host genes, a subsequent SSD duplication gave rise to the MIR208A-MIR208B pair, as outlined in Fig 8. It is interesting to notice that this pair is related to ohno-miRNAs: since MYH7-MYH7B is an ohnolog gene pair, and so is MYH6-MYH7B, MIR208B-MIR499A and MIR208A-MIR499A are found by our analysis to be intragenic ohno-miRNAs. Their mature sequences share a perfectly conserved seed; 3’ arms differ by just three non-seed nucleotides, and miR-499a-5p has only one 7-mer seed mismatch with miR-208a/b, while the flanking regions diverge widely. Despite being considered *bona fide* miRNAs by MirGeneDB, MIR208A and MIR208B are not present in the TarBase network. MIR499A, MIR208A, and MIR208 are part of a set of miRNAs known as “myomiRs” [58], some of which have already been extensively studied. All three of these miRNAs are known to be co-transcribed alongside their host genes [59] and miR-208b and miR-499 are found in both skeletal and cardiac muscle tissue, whereas miR-208a is uniquely expressed in the heart [60,61]. Moreover, miR-208a and miR-208b are known to be chamber-specific, as miR-208a is reported to be abundant in the atrial myocardium, while miR-208b is reported to be preferentially expressed in the left ventricle [62,63]. We argue that this spatial partitioning may mitigate stoichiometric imbalance.

### Robustness of the results

We selected genes and interactions according to a few databases that represent the state-of-the-art in the field: miRNA–target interactions were obtained from the TarBase database, and protein–protein interactions from the PrePPI database. It is interesting to see what happens to our results if we choose different databases for the interactions. We also evaluated the potential impact of literature bias in TarBase—arising from the overrepresentation of targets associated with extensively studied miRNAs—by comparing the results obtained from the entire database with those derived exclusively from high-throughput datasets. In particular, as discussed above, we chose MirDIP as an alternative database for miRNA-target interactions and STRING for protein-protein interactions. As for those used in the main text, these are also state-of-the-art sources, but they select the interactions using criteria that are, so to speak, orthogonal to the previous ones. In fact, as mentioned above, they show a very small number of common interactions. In S1 Text we performed the same analyses discussed in the main text, changing the databases. It turns out that our results, both for the miRNA selection and the enrichment patterns, are essentially the same. We consider this a major test of the robustness of our results. We also performed our analyses on subsets of duplicate miRNA pairs, separating pre-2R duplicates from post-2R duplicates, as well as removing pairs from large duplicate families to reduce possible confounding effects. In all these cases patterns do not change significantly.

## Concluding remarks

The main goal of this paper was to study the role of WGD-derived miRNAs in the human regulatory network. We identified a set of overrepresented regulatory motifs involving these miRNAs, whose specific enrichment is likely due to the dosage balance constraint. We also realized that these ohno-miRNA pairs show a strong tendency to maintain the same seed sequence and regulate common target genes. The combination of these two trends leads to an increase in regulatory redundancy, a hallmark of biological complexity [34]. Indeed, the same pattern was observed when examining WGD pairs of Transcription Factors and their enriched motifs in the transcriptional layer of the regulatory network [11]. Our results could be extended in two directions. First, the same approach used in this paper to identify intragenic ohno-miRNAs could be applied to study other non-coding genes hosted in the introns of (or in proximity to) ohnolog protein-coding genes. In particular, it would be interesting to identify WGD-derived long non-coding RNAs (lncRNAs) and thus prioritize their study. Second, our analysis could be extended using the information contained in [19,20] to distinguish the differences between the miRNAs duplicated in the two different rounds of WGD and to assess whether they play different roles within the regulatory network. Both of these directions are worthwhile to explore further to support the observation, which is the main result of our work: that the two rounds of Whole Genome Duplication played an essential role in increasing the complexity of the regulatory network, which is most likely at the origin of the impressive variety and complexity of the organisms belonging to the vertebrate lineage.

## Supporting information

S1 Text Supplementary material. **Tables A and B.** Putative intragenic ohno- and SSD-derived miRNA pairs without filtering Ensembl duplicates. **Tables C and D.** Intragenic and intergenic miRNAs in GENCODE and MirGeneDB. **Figure A.** Sequence similarity and target similarity OF Ensembl SSD-duplicates. **Figure B.** Out-degree distribution of Ensembl SSD-duplicates. **Figure C.** Target similarities in the MirDIP network. **Figure D.** Out-degree distributions in the MirDIP network. **Figure E.** Motif enrichment analysis in different datasets (Ensembl SSD-duplicates, MirDIP and STRING). **Figure F.** Motif enrichment analysis considering different subsets of the SSD-derived pairs. **Figure G.** Sequence and target similarity of pre- and post-2R SSD-derived pairs. **Figure H.** Distribution of last common ancestors across duplicate pairs. **Figures I and J** Expression of ohno- and SSD-derived miRNAs across multiple human tissues in MirGeneDB and MiRNATissueAtlas. **Tables E and F.** Discarded bifans and PPI-bifans. **Tables G, H, I and J.** Summary of the presence of duplicate protein-coding genes and miRNAs in TarBase and MirDIP. **Tables K and L.** Summary of the presence of duplicate protein-coding genes in PrePPI and STRING. **Figure K.** Sequence similarity of putative intragenic miRNA pairs in the mouse genome. **Tables M and N**. Putative intragenic ohno- and SSD-derived miRNA pairs in the mouse genome. **Figure L.** Sequence similarity of putative intragenic miRNA pairs in three vertebrate genomes (rhesus macaque, brown rat and green anole). **Figure M.** Explicative scheme of the “pairwise” Z-score for motif enrichment.
(PDF)
