# Supplementary material for: Ohno-miRNAs: miRNA pairs derived from whole-genome duplication
Source: PLoS Comput Biol. 2025 Dec 3;21(12):e1013766. doi: 10.1371/journal.pcbi.1013766 (PMC12688097; doi:10.1371/journal.pcbi.1013766)
Supplement: S1 Text — Tables A and B. Putative intragenic ohno- and SSD-derived miRNA pairs without filtering Ensembl duplicates. Tables C and D. Intragenic and intergenic miRNAs in GENCODE and MirGeneDB. Figure A. Sequence similarity and target similarity OF Ensembl SSD-duplicates. Figure B. Out-degree distribution of Ensembl SSD-duplicates. Figure C. Target similarities in the MirDIP network. Figure D. Out-degree distributions in the MirDIP network. Figure E. Motif enrichment analysis in different datasets (Ensembl SSD-duplicates, MirDIP and STRING). Figure F. Motif enrichment analysis considering different subsets of the SSD-derived pairs. Figure G. Sequence and target similarity of pre- and post-2R SSD-derived pairs. Figure H. Distribution of last common ancestors across duplicate pairs. Figures I and J Expression of ohno- and SSD-derived miRNAs across multiple human tissues in MirGeneDB and MiRNATissueAtlas. Tables E and F. Discarded bifans and PPI-bifans. Tables G, H, I and J. Summary of the presence of duplicate protein-coding genes and miRNAs in TarBase and MirDIP. Tables K and L. Summary of the presence of duplicate protein-coding genes in PrePPI and STRING. Figure K. Sequence similarity of putative intragenic miRNA pairs in the mouse genome. Tables M and N. Putative intragenic ohno- and SSD-derived miRNA pairs in the mouse genome. Figure L. Sequence similarity of putative intragenic miRNA pairs in three vertebrate genomes (rhesus macaque, brown rat and green anole). Figure M. Explicative scheme of the “pairwise” Z-score for motif enrichment. (PDF) [file pcbi.1013766.s001.pdf]

SUPPLEMENTARY MATERIAL

Ohno-miRNAs: miRNA pairs derived from whole-genome duplication

Leonardo Agasso[1,*], **Ivan Molineris**[2], **and Michele Caselle**[1]

[1]Department of Physics, University of Turin and INFN, Turin, Italy

[2]Department of Life Sciences and Systems Biology, University of Turin, Turin, Italy

[*]leonardo.agasso@unito.it

1 Putative intragenic miRNA pairs

In Section 1.2 of the Main Text, we identified a list of putative duplicate pairs and kept only those that are recognized as paralogues by Ensembl for further analysis. Here, we report a list that includes all the putative duplicate pairs retrieved by our analysis. The list of all putative pairs, including miRNAs not recognized as *bona fide* by MirGeneDB, is available as a TSV file in the ./lists/ folder of the project GitHub repository linked in the main text.

Table A. Putative intragenic ohno-miRNA pairs without filtering the Ensembl duplicates. Rows highlighted in red are those recognized as duplicates by either Ensembl or MirGeneDB, and thus reported in the Main Text.

Putative Ohno-miRNA 1	Host gene 1	Putative Ohno-miRNA 2	Host gene 2
MIR199A1	DNM2	MIR199B	DNM1
MIR199A1	DNM2	MIR199A2	DNM3
MIR199B	DNM1	MIR199A2	DNM3
MIR103A1	PANK3	MIR103A2	PANK2
MIR103A1	PANK3	MIR107	PANK1
MIR103A2	PANK2	MIR107	PANK1
MIR26B	CTDSP1	MIR26A1	CTDSPL
MIR26B	CTDSP1	MIR26A2	CTDSP2
MIR26A1	CTDSPL	MIR26A2	CTDSP2
MIR196A2	HOXC6	MIR196A1	HOXB7
MIR10A	HOXB3	MIR10B	HOXD3
MIR218-1	SLIT2	MIR218-2	SLIT3
MIR204	TRPM3	MIR211	TRPM1
MIR152	COPZ2	MIR148B	COPZ1
MIR128-1	R3HDM1	MIR128-2	ARPP21
MIR153-1	PTPRN	MIR153-2	PTPRN2
MIR33B	SREBF1	MIR33A	SREBF2
MIR499A	MYH7B	MIR208B	MYH7
MIR499A	MYH7B	MIR208A	MYH6
MIR3613	TRIM13	MIR16-2	TRIM59-IFT80

Table B. **Putative SSD-derived miRNA pairs without filtering the Ensembl duplicates**. Rows highlighted in red are those recognized as duplicates by either Ensembl or MirGeneDB, and thus reported in the Main Text.

Putative paralogue miRNA 1	Host gene 1	Putative paralogue miRNA 2	Host gene 2
MIR208B	MYH7	MIR208A	MYH6
MIR489	CALCR	MIR642A	GIPR
MIR105-2	GABRA3	MIR452	GABRE
MIR105-1	GABRA3	MIR452	GABRE
MIR499A	MYH7B	MIR1266	MYO5C
MIR499A	MYH7B	MIR3136	TMF1
MIR499A	MYH7B	MIR2116	MYO1E
MIRLETF2	HUWE1	MIR140	WWP2
MIR208B	MYH7	MIR1266	MYO5C
MIR208B	MYH7	MIR3136	TMF1
MIR208B	MYH7	MIR2116	MYO1E
MIR208B	MYH7	MIR208A	MYH6
MIR582	PDE4D	MIR139	PDE2A
MIR10A	HOXB3	MIR615	HOXC5
MIR10A	HOXB3	MIR196A2	HOXC6
MIR10A	HOXB3	MIR615	HOXC4
MIR10A	HOXB3	MIR196A1	HOXB7

Putative paralogue miRNA 1	Host gene 1	Putative paralogue miRNA 2	Host gene 2
MIR10B	HOXD3	MIR615	HOXC5
MIR10B	HOXD3	MIR196A2	HOXC6
MIR10B	HOXD3	MIR615	HOXC4
MIR10B	HOXD3	MIR196A1	HOXB7
MIR1266	MYO5C	MIR3136	TMF1
MIR1266	MYO5C	MIR2116	MYO1E
MIR1266	MYO5C	MIR208A	MYH6
MIR561	GULP1	MIR556	NOS1AP
MIR3136	TMF1	MIR2116	MYO1E
MIR3136	TMF1	MIR208A	MYH6
MIR218-1	SLIT2	MIR876	LINGO2
MIR589	FBXL18	MIR887	FBXL7
MIR2116	MYO1E	MIR208A	MYH6
MIR676	EDA	MIR455	COL27A1
MIR101-1	JAK1	MIR151A	PTK2
MIR615	HOXC5	MIR196A2	HOXC6
MIR615	HOXC5	MIR196A1	HOXB7
MIR876	LINGO2	MIR218-2	SLIT3
MIR196A2	HOXC6	MIR615	HOXC4
MIR615	HOXC4	MIR196A1	HOXB7

2 miRNA statistics: intragenic and intergenic miRNAs

In the human genome, miRNAs can be intragenic (hosted on a gene, usually in the introns) or intergenic. As a reference, we summarize here the nature of all the miRNAs from GENCODE. A miRNA is labeled as intragenic if it can be found in the intron (or exon) of at least one protein-coding gene transcript. MiRNAs from MirGeneDB represent a subset of these miRNAs (see Tab.D).

Table C. Intragenic and intergenic miRNAs in GENCODE. Intragenic and intergenic miRNAs among the 1,879 miRNAs in the GENCODE database

N. intragenic miRNAs	N. intergenic miRNAs
1,203 (64%)	676 (36%)

Table D. Intragenic and intergenic miRNAs in MirGeneDB. Intragenic and intergenic miRNAs among the 505 miRNAs in the GENCODE database that are recognized as *bona fide* by MirGeneDB

N. intragenic miRNAs	N. intergenic miRNAs
245 (49%)	260 (51%)

As stated in Section 1.3 of the main text, this fraction of intragenic miRNAs suggests that, when taking gene pairs at random, one would expect to find some putative intragenic duplicate miRNAs by chance. In the human genome, protein coding genes hosting at least one miRNA (according to GENCODE data) are 1,105 out of 20,457(5%). Assuming this distribution is random and that gene pairs are independent, we would expect approximately 307 SSD-derived gene pairs to contain miRNAs in both genes by chance. However, this assumes statistical independence between gene pairs, which does not fully hold for SSD-derived genes, as they often occur in larger families, making this an upper limit. To estimate a lower bound,

we treat each of the 2,210 SSD gene families as a single unit and ask how often two genes in a given family might both host a miRNA. Under the same 5% host frequency, this yields an expected number of approximately 6 pairs of putative intragenic SSD-derived miRNA pairs. The actual number of observed putative intragenic SSD-derived miRNA pairs is 46, which lies well within this interval. This suggests that a portion of these pairs may result from random co-occurrence rather than genuine duplication of miRNAs along with their host genes.

3 Ensembl SSD-derived miRNAs: sequence similarity, target similarity, outdegree distribution

The analyses of sequence similarity, target similarity, and outdegree distribution in the TarBase network have been replicated on SSD-derived miRNAs from Ensembl instead of those derived from MirGeneDB. Ensembl does not explicitly distinguish between duplication mechanisms (WGD vs. SSD) for miRNAs, and its paralogue annotations are inferred without incorporating curated evolutionary context, as MirGeneDB does.

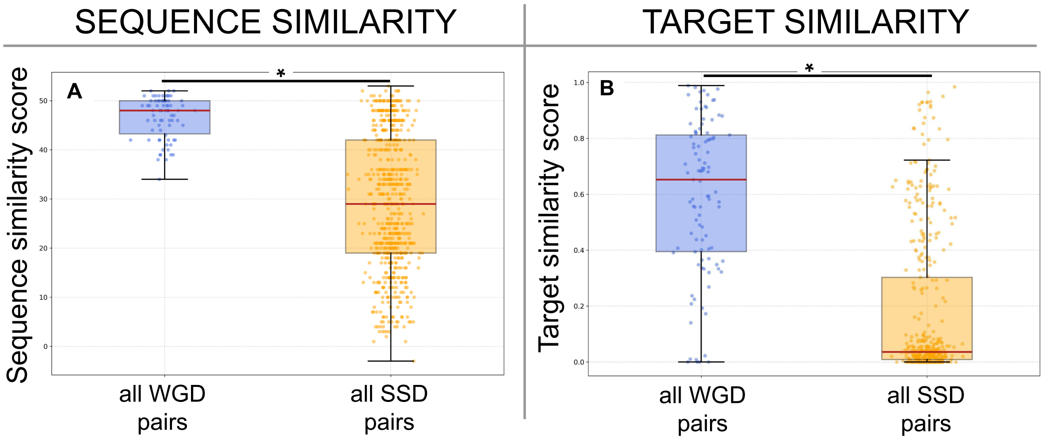

Figure A. Sequence similarity and target similarity of Ensembl SSD-duplicates. Results do not significantly differ from those presented in the Main Text. (*: p<0.01, Kolmogorov-Smirnov test).

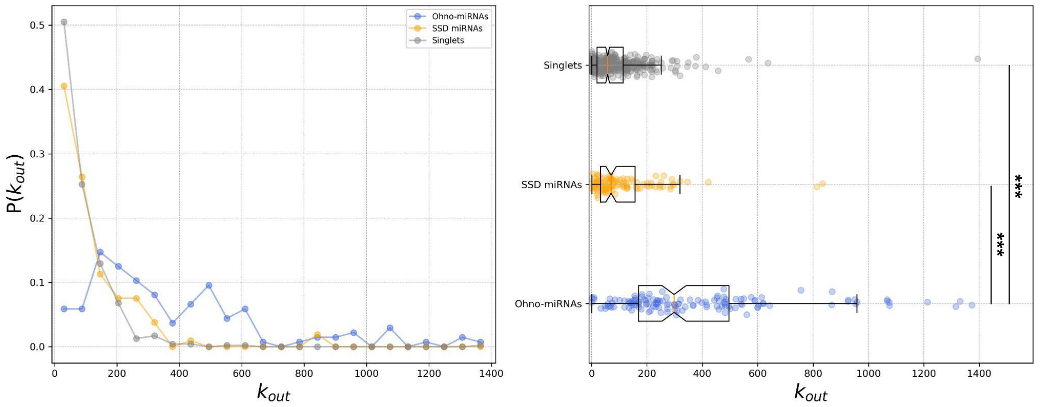

Figure B. Out-degree distribution of Ensembl SSD-duplicates. Out-degree distribution of ohno-miRNAs in the TarBase network, compared with that of the SSD-derived miRNAs from Ensembl, and all the miRNAs in the same network. (***: p<0.001, Kolmogorov-Smirnov test).

4 MirDIP network: target similarity and outdegree distribution

All the analyses in the Main Text conducted on the TarBase network have been replicated on the MirDIP network, keeping only the interactions in the *Very High* score class (which represents the top 1% of interactions). Results do not significantly differ from those presented in the Main Text.

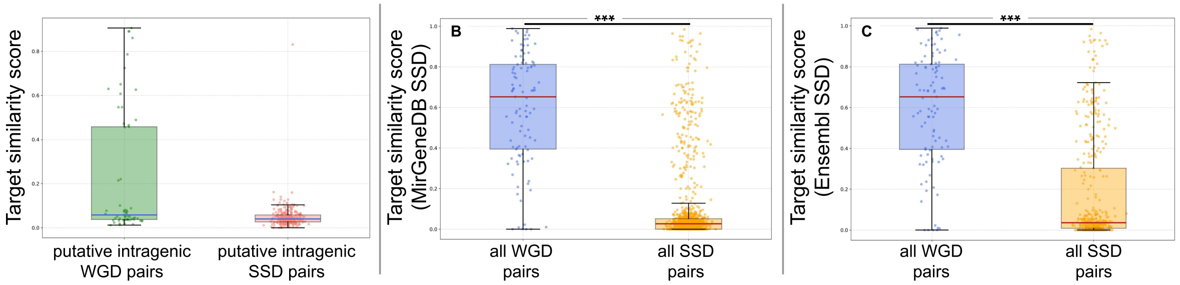

Figure C. Target similarities in the MirDIP network. Results are consistent with those obtained on the TarBase network. (***: p<0.001, Kolmogorov-Smirnov test).

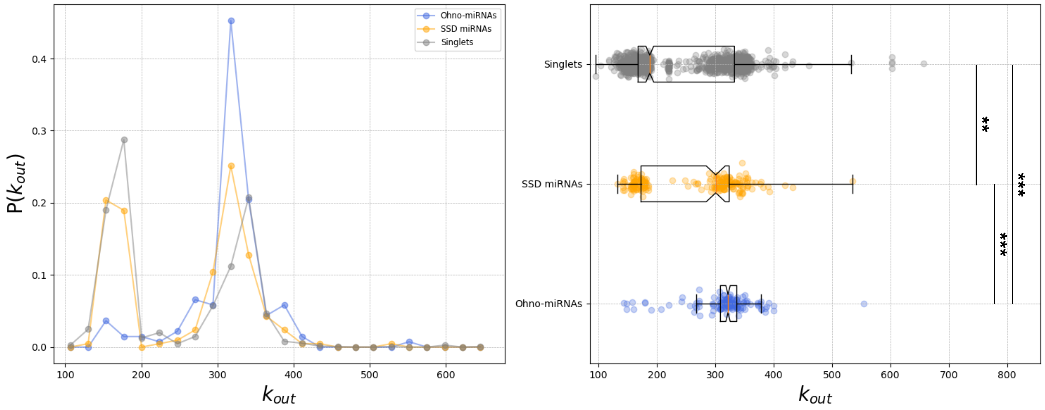

Figure D. **Out-degree distributions in the MirDIP network.** Out-degree distribution of ohno-miRNAs compared with that of the SSD-derived miRNAs from MirGeneDB, and all the miRNAs in the same network. (***: p<0.001, **: p<0.01, Kolmogorov-Smirnov test)

5 Robustness of motif enrichment results

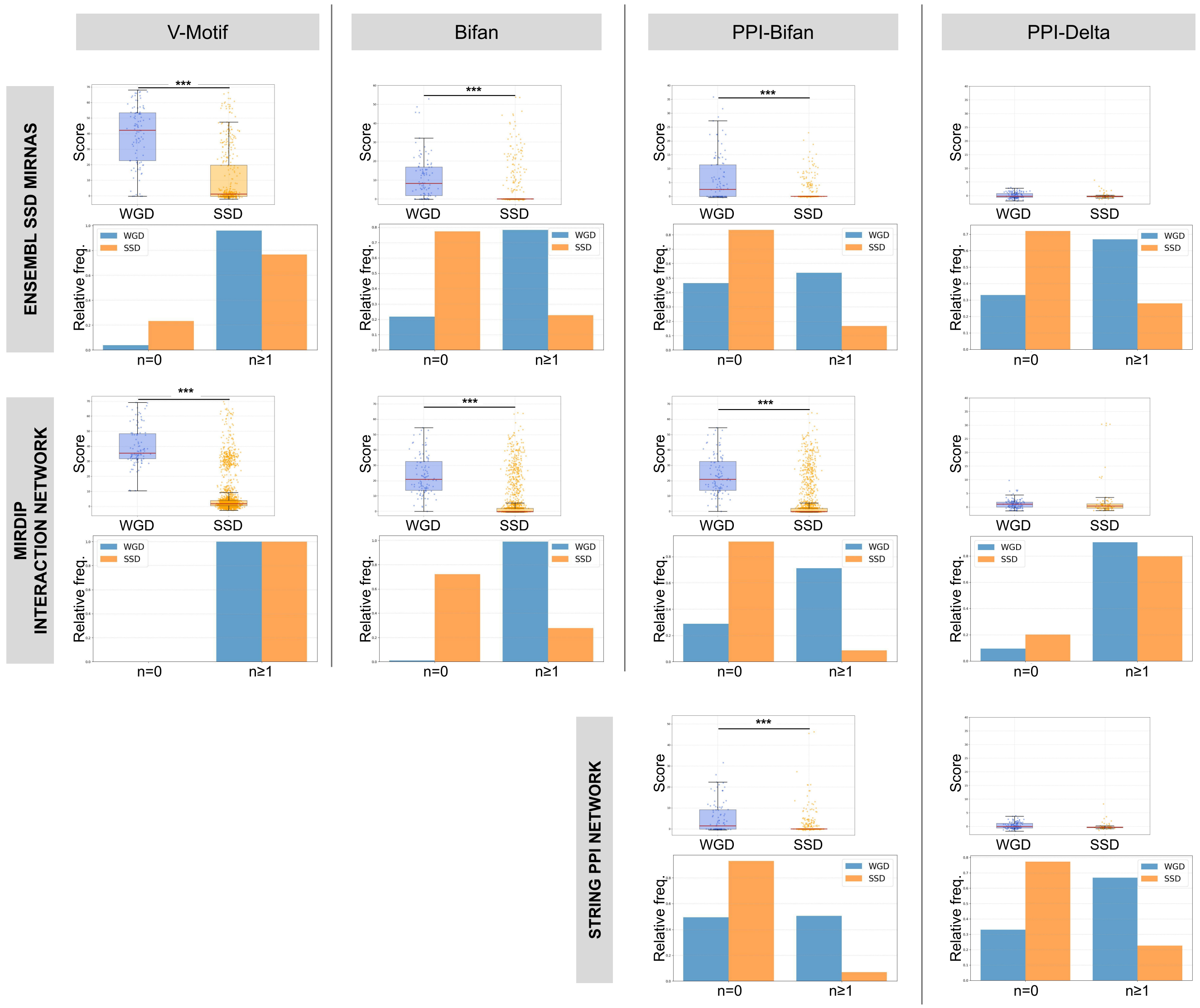

Figure E. Motif enrichment analysis in different datasets (Ensembl SSD-duplicates, MirDIP interaction network and STRING PPI-network). Here we changed SSD-derived miRNA, using pairs retrieved from Ensembl (first row); Interaction network, switching from TarBase to MirDIP (second row); PPI interaction network, switching from PrePPI to STRING (third row). None of these changes has produced results that are in contrast to those presented in the main text. (***: p<0.001, Kolmogorov-Smirnov test)

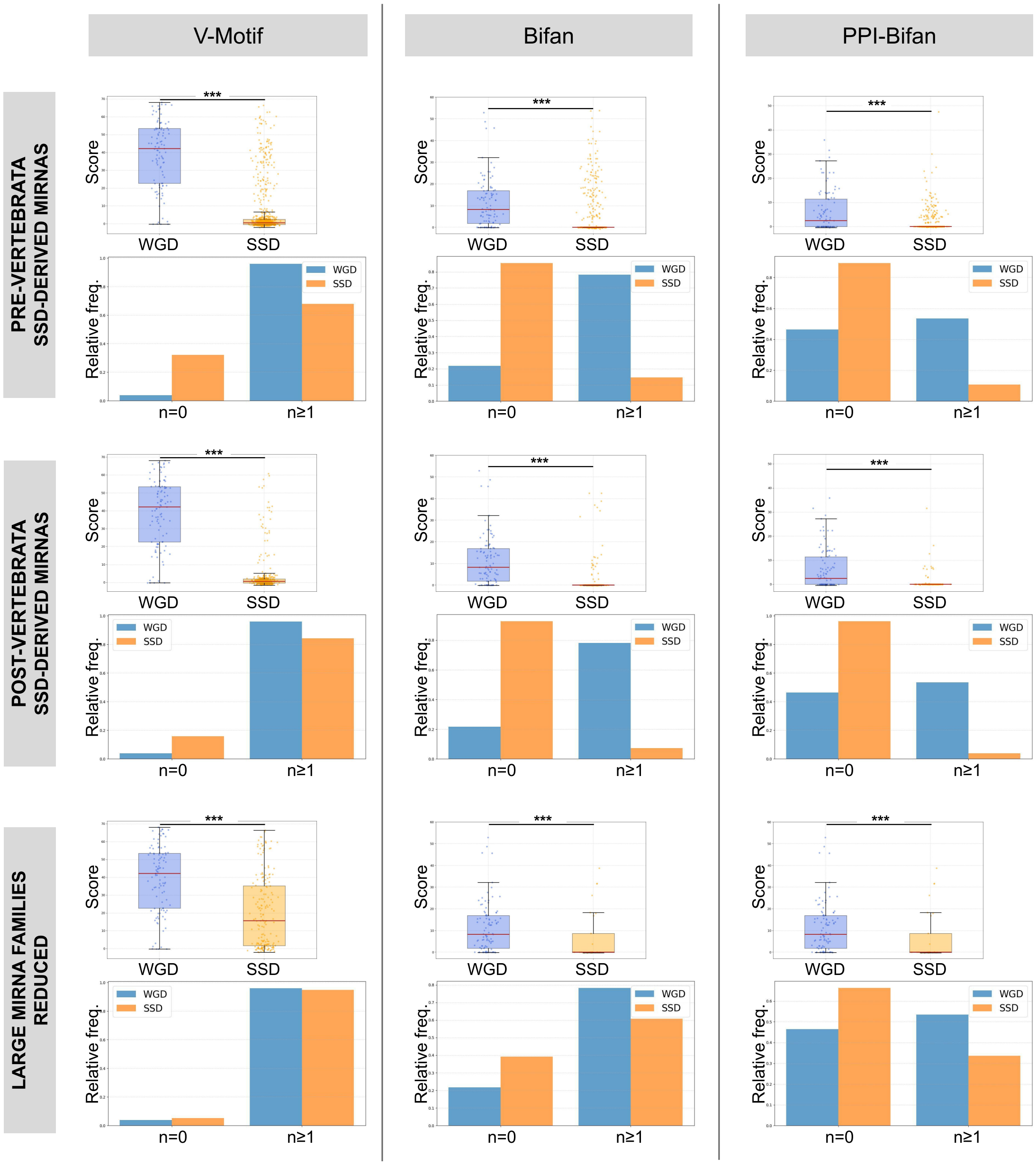

Figure F. **Motif enrichment analysis considering different subsets of the SSD-derived pairs.** Here we considered different subsets of SSD-derived pairs. SSD-pairs whose last common ancestor according to MirGeneDB is older than the two rounds of WGD (i.e. older than, or equal, to "Vertebrata". See Fig H for reference) (first row); SSD-pairs whose last common ancestor according to MirGeneDB is younger than, or equal, to "Vertebrata" (second row); large families (more than 8 pairs) are reduced, keeping only the 8 pairs with the largest Z-score, to mitigate the confounding effect of large duplicate families (third row). (***: $p<0.001$, Kolmogorov-Smirnov test).

6 Sequence and target similarities are lower for recent SSD-derived miRNA pairs

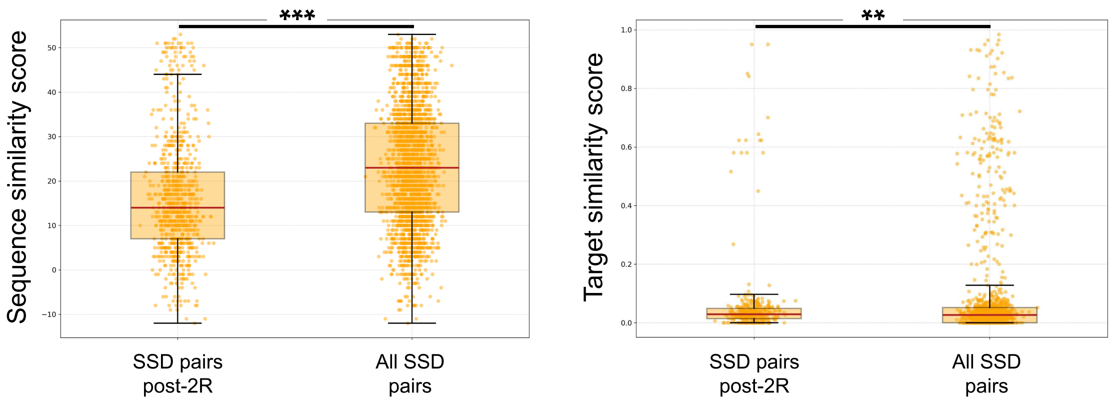

Figure G. Sequence and target similarity of pre- and post-2R SSD-derived pairs. Sequence similarity and target similarity of all SSD-derived miRNA pairs from MirGeneDB, compared with duplicates whose last common ancestor is more recent than the 2 rounds of whole genome duplication (Last common ancestor older than "Gnathostomata"). In both cases the median value is significantly lower when considering more recent duplicates. (**: p<0.01, ***: p<0.001, Kolmogorov-Smirnov test).

7 Distribution of last common ancestor annotations across duplicate miRNA pairs

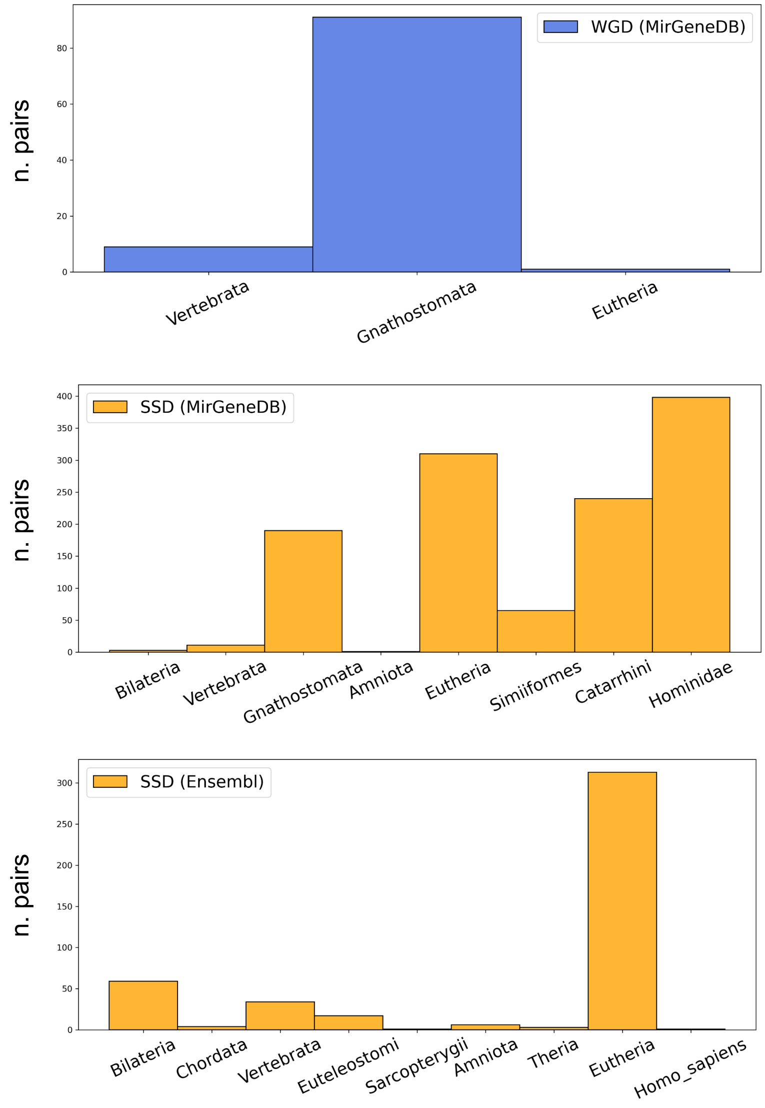

Figure H. Distribution of last common ancestors across duplicate pairs. LCAs are sorted according to their phylogenetic order from most ancient to most recent. When using MirGeneDB, the last common ancestor of a pair is the older between the clades of origin of the two miRNAs.

8 Ohno-miRNA expression on miRNATissueAtlas and MirGeneDB

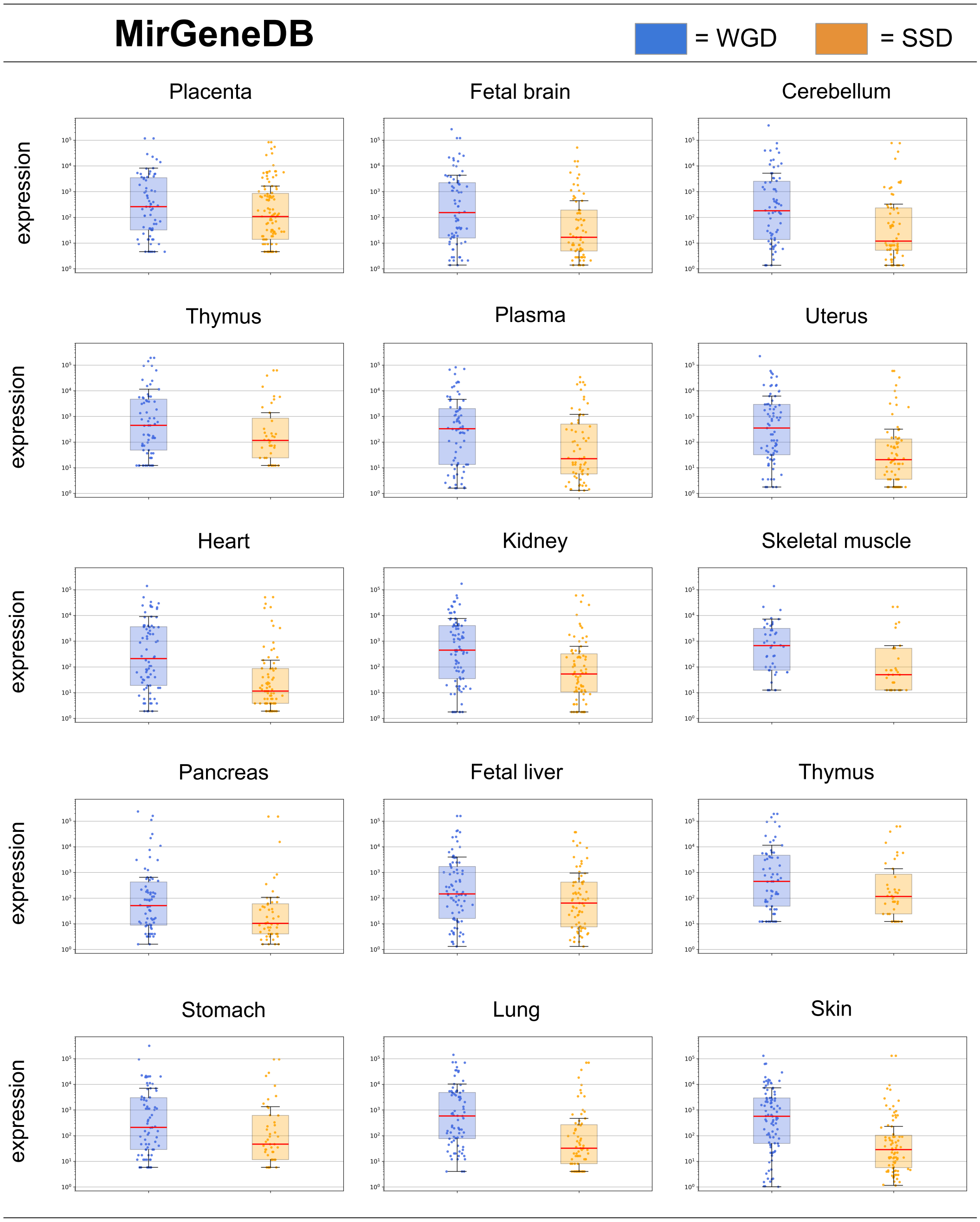

Figure I. Expression of ohno- and SSD-derived miRNAs across multiple human tissues in MirGeneDB. Median expression of ohno-miRNAs is consistently larger than SSD-derived miRNAs.

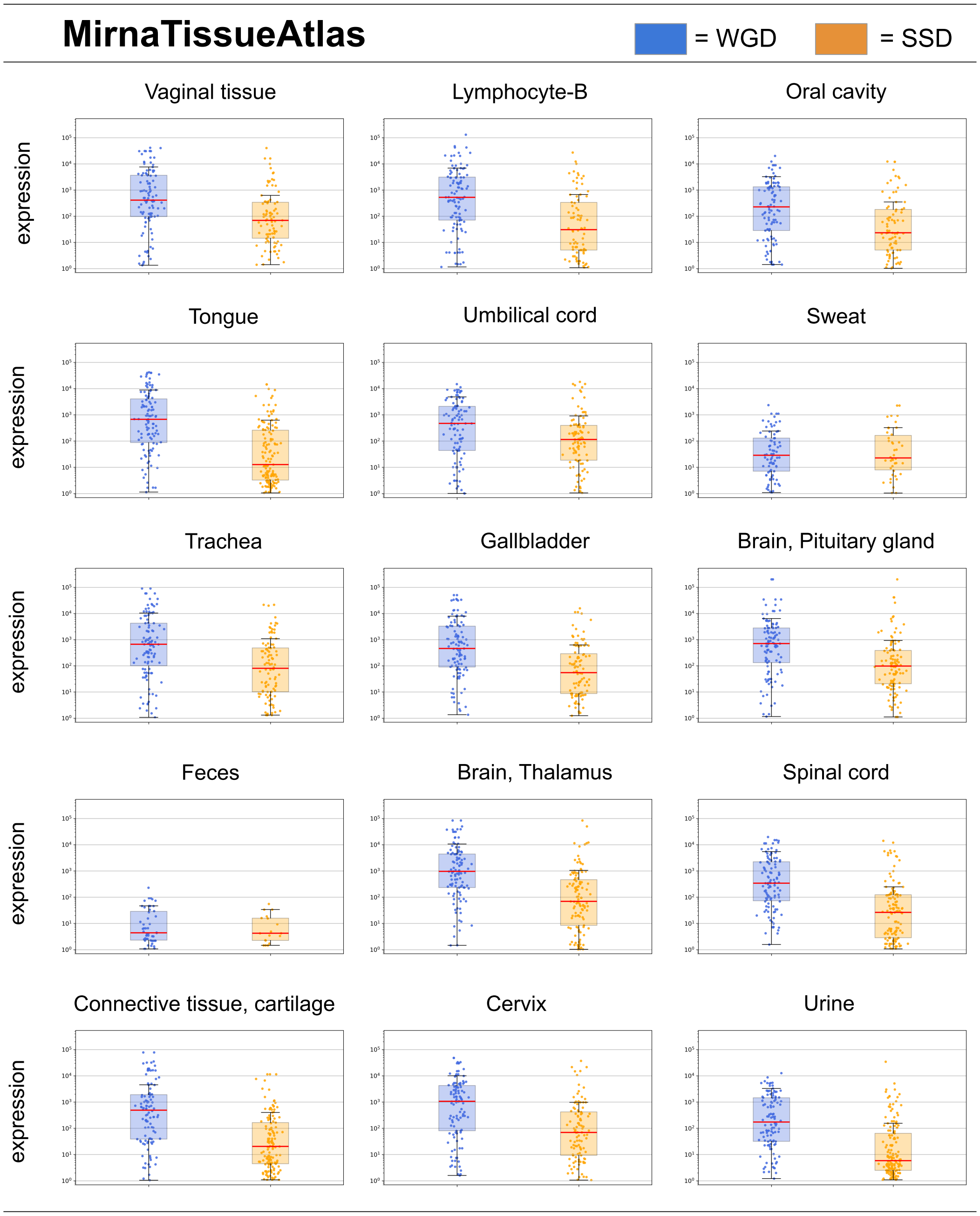

Figure J. Expression of ohno- and SSD-derived miRNAs across multiple human tissues in MiRNATissueAtlas. Median expression of ohno-miRNAs is consistently larger than SSD-derived miRNAs.

9 Discarded pairs from the Z-score plots

When plotting the Z-score for each duplicate miRNA pair, a few pairs are discarded because of the impossibility to compute a Z-score. This happens because, given a number of motifs $n > 0$ observed in the real network, the average value $\overline{n}_{null}$ resulted in being equal to 0, and so also $\sigma_{null} = 0$. For the sake of completeness, we report here the pairs discarded from the results in the Main Text, and with different combinations of interaction networks and PPI networks, together with the number of motifs (n) they are involved in the real network.

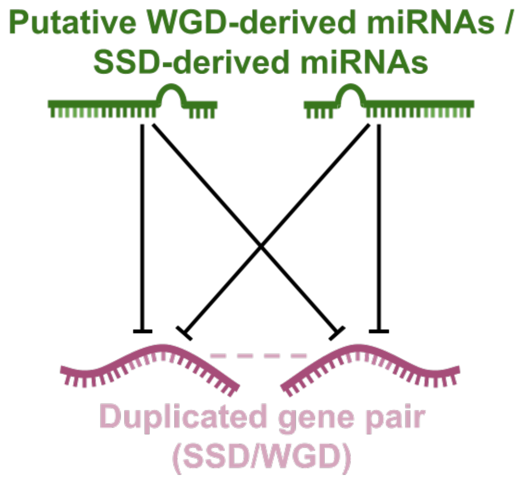

**A.** TarBase		
Pair	Set	n
MIR1-1 - MIR1-2	*SSD* (MirGeneDB, Ensembl)	1
MIR302A - MIR518B	*SSD* (MirGeneDB, Ensembl)	1
MIR302B - MIR518B	*SSD* (MirGeneDB)	1
MIR302C - MIR518D	*SSD* (MirGeneDB)	1
MIR302C - MIR518B	*SSD* (MirGeneDB)	1
MIR302D - MIR518B	*SSD* (MirGeneDB)	1
MIR516B1 - MIR516B2	*SSD* (MirGeneDB)	2
MIR518B - MIR518D	*SSD* (MirGeneDB, Ensembl)	1
MIR521-1 - MIR521-2	*SSD* (MirGeneDB)	1
MIR320C1 - MIR320C2	*SSD* (MirGeneDB)	1
MIR548F1 - MIR548F2	*SSD* (Ensembl)	1

Table E. Discarded bifans. Discarded pairs, with respective number n of motifs, from the analysis of simple bifan motif.

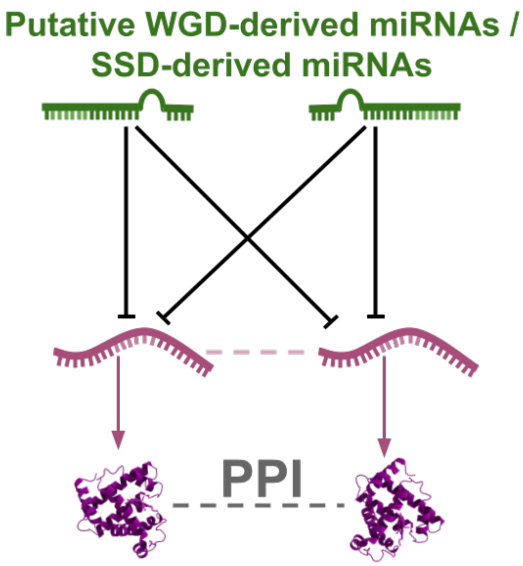

**B.** TarBase + PrePPI		
Pair	Set	*n*
MIR301A - MIR301B	*WGD*	2
MIR18A - MIR18B	*WGD*	1
MIR100 - MIR99A	*SSD* (MirGeneDB, Ensembl)	1

**C.** TarBase + STRING		
Pair	Set	*n*
MIR301A - MIR301B	*WGD*	2
MIR181B1 - MIR181D	*WGD*	1
MIR128-1 - MIR128-2	*WGD*	1

**D.** MirDIP + STRING		
Pair	Set	*n*
MIR1-2 - MIR206	*WGD*	2
MIR7-1 - MIR7-3	*WGD*	1
MIR190A - MIR190B	*WGD*	1
MIR133A1 - MIR133B	*WGD*	1
MIR526A1 - MIR526A2	*SSD* (MirGeneDB)	1
MIR519B - MIR526A2	*SSD* (MirGeneDB)	1
MIR320B2 - MIR320C2	*SSD* (MirGeneDB, Ensembl)	1
MIR1283-1 - MIR1283-2	*SSD* (MirGeneDB)	1

MIR320B1 - MIR320B2	*SSD* (MirGeneDB, Ensembl)	1
MIR1184-3 - MIR1184-2	*SSD* (Ensembl)	2
MIR1184-1 - MIR1184-2	*SSD* (Ensembl)	2
MIR941-3 - MIR941-5	*SSD* (Ensembl)	1
MIR941-2 - MIR941-5	*SSD* (Ensembl)	1
MIR941-2 - MIR941-4	*SSD* (Ensembl)	1
MIR941-2 - MIR941-3	*SSD* (Ensembl)	1
MIR8071-1 - MIR8071-2	*SSD* (Ensembl)	1
MIR548AJ1 - MIR548X	*SSD* (Ensembl)	1
MIR378B - MIR378D2	*SSD* (Ensembl)	1
MIR320C2 - MIR320D2	*SSD* (Ensembl)	1
MIR320B2 - MIR320D2	*SSD* (Ensembl)	1
MIR320B1 - MIR320D2	*SSD* (Ensembl)	1

Table F. Discarded PPI-bifans. Discarded pairs, with respective number *n* of motifs, from the analysis of PPI-bifan motif.

10 Summary of the presence of duplicate protein-coding genes and miRNAs in different networks

The presence of a miRNA/gene of interest in a given miRNA-gene interaction network or a protein-protein interaction network is not guaranteed because many factors (biological, technical, etc.) may lead to the absence of a certain number of elements. We provide here a summary of the presence of duplicate miRNAs and genes in all the networks employed in the Main Text and the S1 Text. Individual genes and individual miRNAs from different sets may overlap.

10.1 miRNA-target interaction networks: TarBase and MirDIP

	Total	TarBase	MirDIP
Ohno-miRNAs (*WGD*)	137	136 (99%)	137 (100%)
MirGeneDB paralogue miRNAs (*SSD*)	304	263 (87%)	303 (100%)
Ensembl paralogue miRNAs (*SSD*)	327	219 (67%)	325 (99%)

Table G. Single miRNAs in TarBase and MirDIP. Number and percentage of single relevant miRNAs present in different interaction networks.

	Total	TarBase	MirDIP
Ohno-miRNA pairs (*WGD*)	114	113 (99%)	114 (99%)
MirGeneDB paralogue miRNA pairs (*SSD*)	2,480	1,384 (56%)	2,455 (99%)
Ensembl paralogue miRNA pairs (*SSD*)	1,131	537 (47%)	1,128 (100%)

Table H. Duplicate miRNA pairs in TarBase and MirDIP. Number and percentage of relevant miRNA pairs present in different interaction networks. A pair is counted as present only if both miRNAs are present in the interaction network.

	Total	TarBase	MirDIP
Ohnolog genes (*WGD*)	7,776	4,480 (58%)	6,915 (98%)
SSD-derived genes (*SSD*)	14,054	6,583 (48%)	10,941 (79%)

Table I. Single protein-coding genes in TarBase and MirDIP. Number and percentage of single relevant protein-coding genes present in different interaction networks.

	Total	TarBase	MirDIP
Ohnolog gene pairs (*WGD*)	9,870	2,982 (32%)	7,482 (80%)
SSD-derived gene pairs (*SSD*)	132,003	29,008 (24%)	71,713 (581%)

Table J. Duplicate protein-coding gene pairs in TarBase and MirDIP. Number and percentage of relevant protein-coding gene pairs present in different interaction networks. A pair is counted as present in an interaction network only if both genes are present.

10.2 Protein-protein interaction networks: PrePPI and STRING

	Total	PrePPI	STRING
Ohnolog genes (*WGD*)	7,776	4,364 (56%)	4,151 (53%)
SSD-derived genes (*SSD*)	14,054	6,481 (46%)	6,729 (48%)

Table K. Single protein-coding genes in PrePPI and STRING. Number and percentage of single relevant protein-coding genes present in different protein-protein interaction networks.

	Total	PrePPI	STRING
Ohnolog gene pairs (*WGD*)	9,870	3,491 (35%)	3,166 (32%)
SSD-derived gene pairs (*SSD*)	132,003	31,485 (24%)	23,805 (18%)

Table L. Duplicate protein-coding gene pairs in PrePPI and STRING. Number and percentage of relevant protein-coding gene pairs present in different protein-protein interaction networks. A pair is counted as present in an interaction network only if both genes are present.

11 Putative intragenic ohno-miRNAs in the mouse genome

Here we report the lists of the putative intragenic ohnolog miRNAs in the mouse genome, together with the analysis of their sequence similarity (Fig K). Pairs highlighted in red are recognized as duplicated pairs by either Ensembl or MirGeneDB.

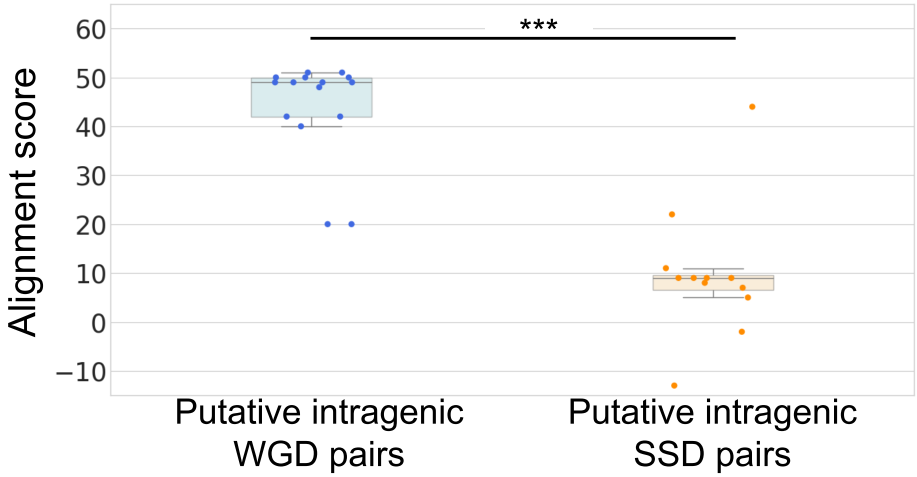

Figure K. Sequence similarity of putative intragenic miRNA pairs in the mouse genome.

Comparison of alignment scores (miRNA sequence similarity) for WGD and SSD pairs for intragenic miRNA pairs detected in the mouse genome following the same pipeline used for the human genome. Results are consistent with the patterns observed in the human genome. (***: $p<0.001$, Kolmogorov-Smirnov test).

Table M. **Putative intragenic ohno-miRNA pairs in the mouse genome.**

Putative Ohno-miRNA 1	Host gene 1	Putative Ohno-miRNA 2	Host gene 2
Mir103-1	Pank3	Mir103-2	Pank2
Mir26b	Ctdsp1	Mir26a-1	Ctdspl
Mir26b	Ctdsp1	Mir26a-2	Ctdsp2
Mir26a-1	Ctdspl	Mir26a-2	Ctdsp2
Mir199b	Dnm1	Mir199a-1	Dnm2
Mir199b	Dnm1	Mir199a-2	Dnm3
Mir1991-1	Dnm2	Mir199a-2	Dnm3
Mir218-1	Slit2	Mir218-1	Slit3
Mir152	Copz2	Mir148b	Copz1
Mir103-1	Pank3	Mir107	Pank2
Mir103-2	Pank2	Mir107	Pank1
Mir211	Trpm1	Mir204	Trpm3
Mir128-2	Arpp21	Mir128-1	R3hdm1
Mir208a	Myh6	Mir499	Myh7b
Mir208b	Myh7	Mir499	Myh7b

Table N. **Putative intragenic SSD-derived miRNA pairs in the mouse genome.**

Putative SSD-derived miRNA 1	Host gene 1	Putative SSD-derived miRNA 2	Host gene 2
Mir208a	Myh6	Mir208b	Myh7
Mir452	Gabre	Mir105	Gabra3
Mir582	Pde4d	Mir139	Pde2a
Mir873b	Lingo2	Mir218-2	Slit3
Mir873b	Lingo2	Mir218-1	Slit2
Mirlet7f-2	Huwe1	Mir140-1	Wwp2
Mir455	Col27a1	Mir676-1	Eda
Mir3060	Rnf215	Mir340	Rnf130
Mir196a-2	Hoxc5	Mir10b	Hoxd4
Mir1962	Ptpre	Mir153	Ptprn2

Some pairs are obviously missing, their absence is attributable to wrong annotations in the different genomes. To cite an example, let's consider the pair MIR10A-MIR10B in the human genome, recognized by our analysis as a putative ohnolog pair. Both MIR10A and MIR10B have orthologues in the mouse genome (Mir10a and Mir10b) within the Hox cluster. However, Mir10a is not reported to be intragenic; it's instead located less than 10,000 bases upstream of the Hoxb3 gene; conversely, Mir10b is correctly reported as hosted on Hoxd3. Some pairs are correctly missing, as the well-known MIR33A-MIR33B pair. While Mir33 in the mouse genome is correctly located within the Srebf2 gene, the Srebf1 lacks the orthologue of MIR33B. However, there are many vertebrates whose genomes conserve both the orthologues of MIR33A and MIR33B; for example, the Green Anole (*Anolis Carolinensis*) genome preserves both miRNAs, despite being phylogenetically more distant from the human genome than the

mouse.

The analysis concerning the putative intragenic pairs can be reproduced on other vertebrate genomes. When applying the same sequence alignment approach, we observe similar patterns: putative intragenic WGD-derived pairs tend to be more conserved in sequence than their SSD-derived counterparts. However, due to the limited availability of well-annotated WGD- and SSD-derived protein-coding gene pairs in species beyond human and mouse, the number of retrieved miRNA pairs is very low, making further comparative analyses currently uninformative.

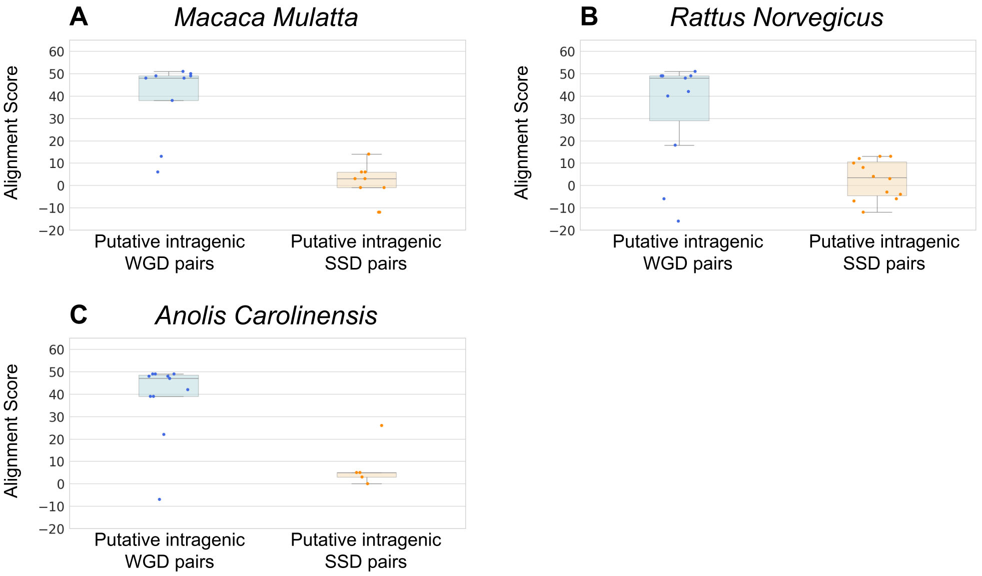

Figure L. Sequence similarity of putative intragenic miRNA pairs in three vertebrate genomes. Results of the alignment of putative intragenic WGD-derived miRNA pairs nd SSD-derived miRNA pairs in the genome of three vertebrates (A, rhesus macaque; B, brown rat; C, green anole). Alignment parameters are the same as in the Main Text.

86
11.1 Quantifying ohnolog miRNA enrichment in different network motifs: pairwise Z-score

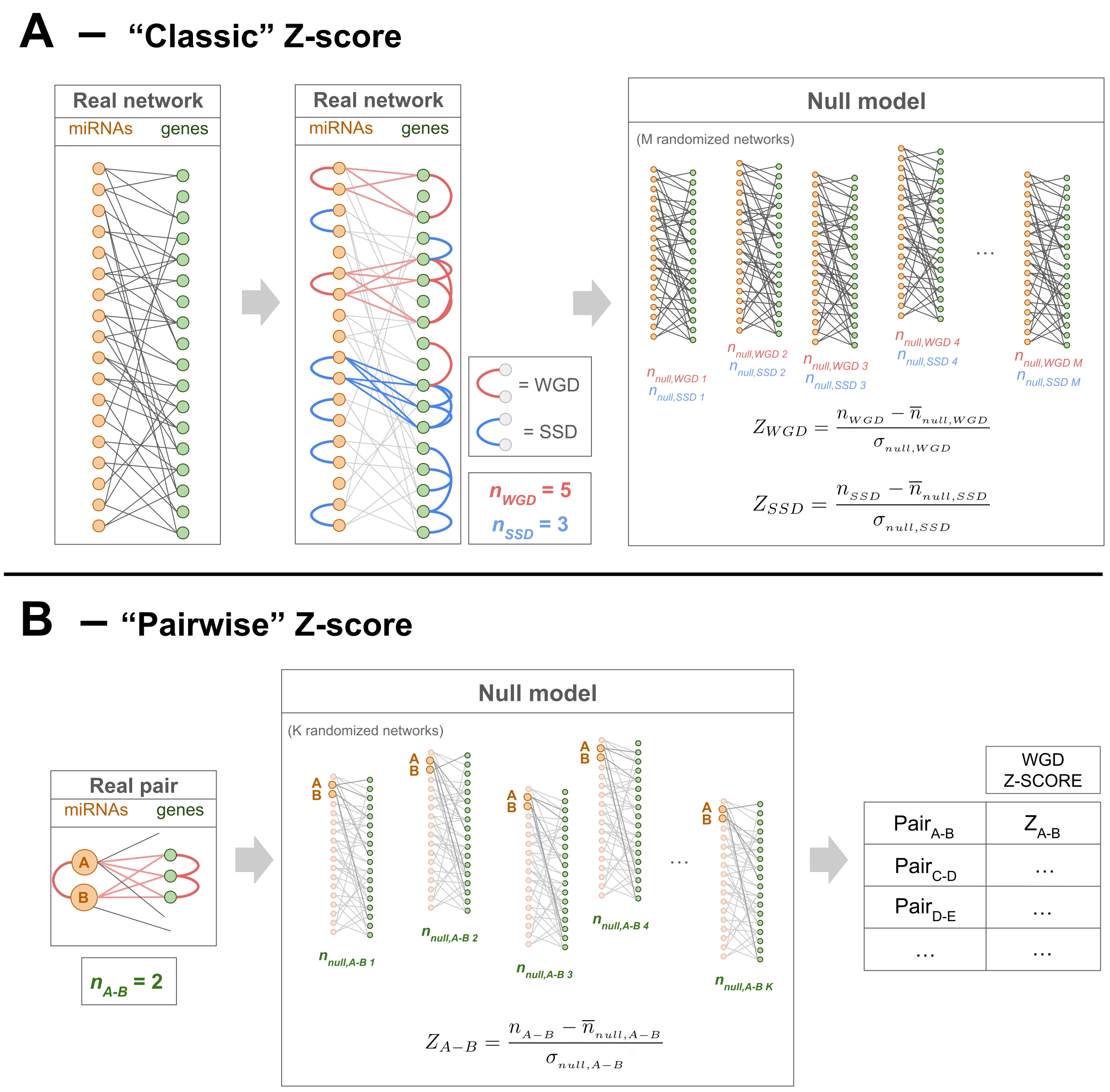

Figure M. Explicative scheme of the "pairwise" Z-score for motif enrichment. Differences between the "classic" and a "pairwise" way of reporting the Z-scores for subset of nodes in a given network (the example considers bifans). In case **(A)** we sum the counts from all the pairs belonging to the same set (e.g. SSD or WGD), thus not knowing if the resulting Z-score is due to all the pairs in the set or just to a subset. On the other hand, in case **(B)** we count the number of motif and generate a distribution of counts over the null model for each pair in each set, obtaining a distribution of Z-scores for each set (WGD, SSD and Ancient SSD). Pairwise Z-score can be straightforwardly extended to single miRNAs when analyzing delta motifs.
